# A Systematic Review and Meta-Analysis of Type 2 Diabetes Prevention Through Lifestyle Interventions in Women with a History of Gestational Diabetes—A Summary of Participant and Intervention Characteristics

**DOI:** 10.3390/nu16244413

**Published:** 2024-12-23

**Authors:** Gebresilasea Gendisha Ukke, Jacqueline A. Boyle, Ahmed Reja, Wai Kit Lee, Mingling Chen, Michelle Shi Min Ko, Chelsea Alycia, Jane Kwon, Siew Lim

**Affiliations:** 1 Health Systems and Equity, Eastern Health Clinical School, Monash University, Level 2, 5 Arnold Street, Box Hill, VIC 3128, Australia; gebresilasea.ukke@monash.edu (G.G.U.); jacqueline.boyle@monash.edu (J.A.B.); 2 School of Medicine, College of Health Sciences, Addis Ababa University, Addis Ababa 9086, Ethiopia; ahmedreja@yahoo.com; 3 Singapore Health Services, 31 Third Hospital Avenue, Singapore 168753, Singapore; waikit.lee@mohh.com.sg (W.K.L.); michelle.ko.shi.min@gmail.com (M.S.M.K.); 4 Monash Centre for Health Research and Implementation, Monash University, Level 1, 43-51 Kanooka Grove, Clayton, VIC 3168, Australia; milly2012@139.com; 5 Department of Endocrine and Metabolic Diseases, Shanghai Institute of Endocrine and Metabolic Diseases, Ruijin Hospital, Shanghai Jiao Tong University School of Medicine, 197 Ruijin 2nd Road, Shanghai 200025, China; 6 Shanghai National Clinical Research Centre for Metabolic Diseases, Key Laboratory for Endocrine and Metabolic Diseases of the National Health Commission of the PR China, Shanghai Key Laboratory for Endocrine Tumor, Ruijin Hospital, Shanghai Jiao Tong University School of Medicine, Shanghai 200025, China; 7 Faculty of Medicine, Nursing and Health Sciences, Monash University, 264 Ferntree Gully Rd, 14, Notting Hill, VIC 3168, Australia; chelseaalycia18@gmail.com; 8 Diabetes Victoria, Suite G01/15-31 Pelham St., Carlton, VIC 3053, Australia; jkwon@diabetesvic.org.au

**Keywords:** gestational diabetes, intervention characteristics, lifestyle intervention, meta-analysis, tidier, type 2 diabetes

## Abstract

**Objectives**: We aimed to review the effect of lifestyle interventions in women with a history of gestational diabetes mellitus (GDM) based on the participants and intervention characteristics. **Methods**: We systematically searched seven databases for RCTs of lifestyle interventions published up to 24 July 2024. We included 30 studies that reported the incidence of type 2 diabetes mellitus (T2DM) or body weight. A random effects model was used to calculate the relative risk and mean difference with a 95% confidence interval. Subgroup analyses were conducted for participants’ characteristics (age, body mass index (BMI)) and intervention characteristics according to the Template for Intervention Description and Replication (TIDieR). **Results**: A greater T2DM risk reduction was seen in trials that started within one year postpartum, in participants with a mean baseline BMI of 30 kg/m^2^ or more, or based on behavioral change theory. For body weight reduction, studies on participants with a mean baseline BMI of 25 kg/m^2^ or more or ones that included electronic/mobile delivery (text message, web, phone call) were more effective. **Conclusions**: Diabetes prevention trials in women with a history of GDM are more effective when commencing within one year postpartum, underpinned by behavior change theory, and in participants with overweight or obesity.

## 1. Introduction

Diabetes is one of the most significant global health problems in recent decades [1]. In 2021, diabetes affected 537 million adults globally, with 90% of these being type 2 diabetes (T2DM) [1,2]. One of the risk factors for T2DM is gestational diabetes mellitus (GDM), which increases the risk by seven to tenfold for women [3,4]. Lifestyle interventions effectively prevent T2DM in at-risk populations, with an up to 58% risk reduction demonstrated in the United States Diabetes Prevention Program study and the Finnish Diabetes Prevention Study [5,6]. Unlike in the general population who are at risk for T2DM, the effectiveness of lifestyle interventions in preventing T2DM in women with a history of GDM is inconsistent [7,8,9,10,11,12], ranging from no beneficial effect [9,10] to a 43% risk reduction [11]. This variation could be due to participant or intervention characteristics [13].

Intervention characteristics like the intervention type (e.g., diet with physical activity) and provider (e.g., delivered by a health professional) have been demonstrated to have a significant role in the effect size of postpartum weight loss with lifestyle interventions in the general population [14]. In our previous meta-analysis on T2DM prevention in the general population, intervention characteristics such as a higher number of sessions were found to be associated with significantly greater diabetes risk reduction [15]. Other intervention characteristics that are relevant to T2DM prevention in women with a history of GDM and intervention commencement time may also moderate the intervention effectiveness [10,11]. In addition to intervention characteristics, participants’ characteristics, such as age, have also been shown to determine the effectiveness of diabetes prevention programmes [16]. The intervention effectiveness can be optimized by identifying the most effective intervention delivery methods and those who are most likely to benefit from the interventions.

Intervention characteristics can be systematically investigated using the Template for Intervention Description and Replication (TIDieR) checklist [17]. Originally developed as a publication guide, the TIDieR is a checklist of the minimum recommended items for describing intervention characteristics, which include the theory or rationale underpinning the intervention, materials used, intervention provider, mode of delivery, intervention location, number of sessions, intervention tailoring, modifications, and fidelity measures [17]. As such, it is a comprehensive framework that outlines the essential components of an intervention. While some participant characteristics, such as age, and intervention characteristics, such as the number of sessions, have been shown to influence the intervention effect size [10,11,15,16], a comprehensive investigation of these characteristics has not been conducted.

Therefore, this study aimed to systematically review the effect of participant and intervention characteristics according to the TIDieR checklist on the lifestyle interventions (diet, physical activity, or combined) outcomes (T2DM and body weight) in women with a history of GDM.

## 2. Materials and Methods

### 2.1. Data Sources and Searches

The data source and searches of this systematic review and meta-analysis are published elsewhere [18]. By adhering to the Preferred Reporting Items for Systematic Reviews and Meta-Analyses (PRISMA) guidelines [19], we searched MEDLINE, CINAHL, EMBASE, PubMed, PsycINFO, Web of Science, and EBM Reviews, including ACP Journal Club, Cochrane Database of Systematic Reviews, Cochrane Clinical Answers, Cochrane Methodology Register Database of Abstracts of Reviews of Effects, Health Technology Assessment, and NHS Economic Evaluation Database. We used both Medical Subject Headings (MeSH) terms and free key terms (gestational diabetes, lifestyle intervention, physical activity, behaviour, diet, and type 2 diabetes) for the search strategy. In addition, we looked through the references of the included articles and systematic reviews and searched the International Clinical Trial Registry Platform to see if any additional studies could be added. This updated search included all studies that had been published up to 24 July 2024. There were no language restrictions, and we looked for translations whenever possible (Table S1). The protocol was prospectively registered on PROSPERO (CRD42022314231).

### 2.2. Study Selection

For this secondary analysis, we included randomized controlled trials (RCTs) involving diet, physical activity, or behavioural interventions and reported the incidence of T2DM or body weight as an outcome. Included studies were those with interventions that started during pregnancy and continued after childbirth or postpartum interventions among women with a history of GDM at any time. We excluded studies that involved women with type 1 diabetes or T2DM, those that involved supplementation or pharmacological interventions, pregnancy-only interventions that did not continue after birth, those that combined lifestyle intervention with supplementation or pharmacological interventions, and those that did not report T2DM or body weight as an outcome. In addition, we excluded editorials, narrative reviews, conference abstracts, letters, systematic reviews, non-RCTs, single-arm studies, trial registries, commentaries, study protocols, or dissertations. Two reviewers (G.G.U. and M.C. or J.K.) independently screened all the articles for titles/abstracts and full texts and resolved conflicts by consensus or with an arbitrator (S.L.).

### 2.3. Risk of Bias and Quality Assessment

Two independent reviewers (G.G.U. and M.C. or M.S.M.K.) assessed the methodological quality of the included studies and resolved any discrepancies by consensus. We assessed the risk of bias of the included studies using the Cochrane Collaboration Tool for Assessing Risk of Bias tool for Randomized Trials: version 2 (RoB 2) [20]. This tool assesses the risk of bias based on five domains: the randomization process, deviations from intended interventions, missing outcome data, measurement of the outcome, and selection of the reported result. An additional domain was assessed for cluster RCTs, on bias arising from the timing of identification or recruitment of participants within clusters. The risk of bias in each study was rated as low risk, some concerns, and high risk.

### 2.4. Data Extraction

Two reviewers (G.G.U and W.L. or C.A.) independently extracted the data using a standard data form that was prepared for this purpose. The extracted data included study characteristics (author, publication year, study country, study setting, intervention commencement time, sample size), baseline age and weight of participants, and study outcomes (T2DM and body weight). In addition, we extracted intervention characteristics according to the TIDieR checklist: the name of the intervention, use of theory underpinning the intervention, intervention type, intervention description, intervention duration, intervention provider, intervention delivery mode, intervention location, intervention tailoring, intervention modification, number of sessions, and fidelity measures. We contacted the corresponding authors for any missing data. Discrepancies were resolved through consensus (G.G.U. and W.L. or C.A.) or with an arbitrator if required (S.L.).

### 2.5. Data Synthesis and Analysis

Findings were statistically summarized in meta-analyses and checked for publication biases. Subgroup analyses of intervention effects on the T2DM and body weight were carried out based on the intervention characteristics and using the TIDieR framework, baseline mean BMI, baseline mean age, and intervention commencement time. For the incidence of T2DM, we calculated the risk ratio (RR) with a 95% CI using the random effects model and DerSimonian–Laird (DL) estimator. For body weight, we calculated mean differences (MDs) with a 95 percent confidence interval (CI) using the random-effects model with the DL estimator [21]. Statistical heterogeneity was determined by the I^2^ test, with I^2^ values of more than 50% indicating moderate to high heterogeneity [22]. *p* < 0.05 was considered statistical significance. The findings of the meta-analysis and publication bias analyses were displayed using forest plots and the Egger test, respectively. Statistical analyses were conducted using the Stata Statistical Software: Release 16. College Station, TX, USA: StataCorp LLC [23].

## 3. Results

### 3.1. Retrieval and Screening

**Figure 1:**
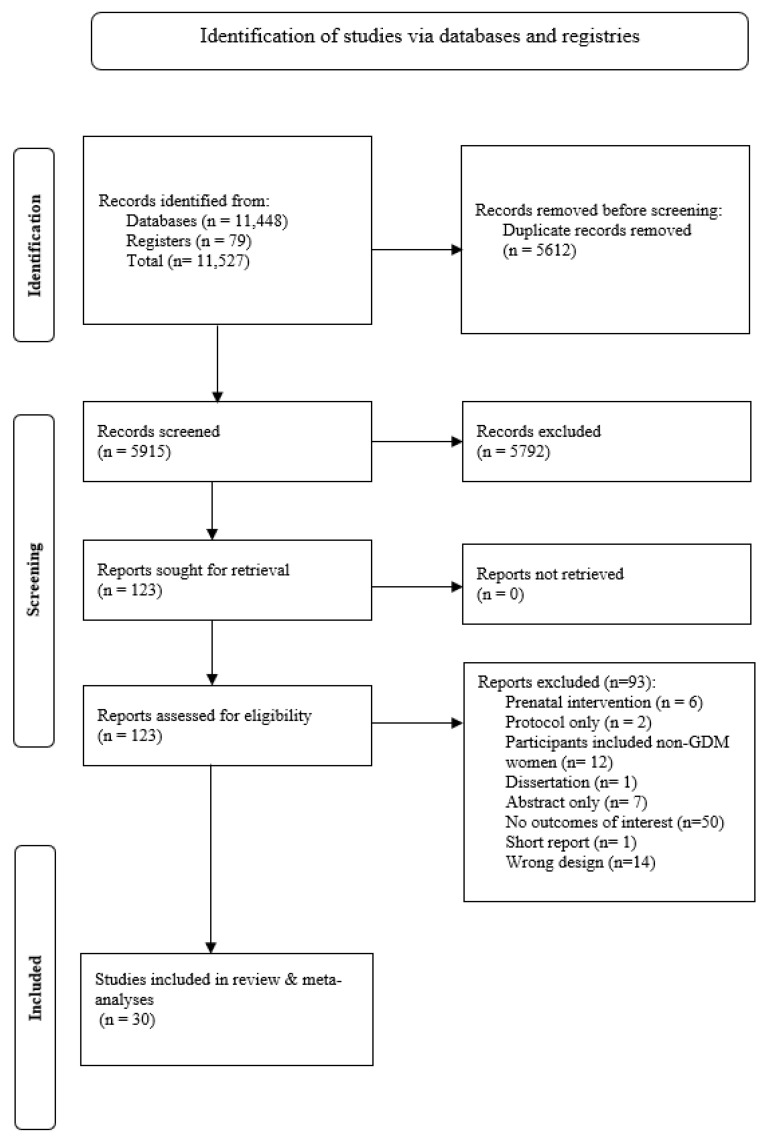
PRISMA diagram of included studies.

A total of 11,527 articles were identified (Figure 1). After removing 5612 duplicates, we screened the title and abstract of 5915 articles, and 123 articles were eligible for full-text screening. We excluded 93 articles during the full-text screening for the following reasons: pregnancy-only interventions, protocols, participants included women without a history of GDM, dissertation, abstracts, no outcomes of interest, a short report, and observational studies/wrong design. Finally, we included 30 articles (16 reported on T2DM and 23 reported on body weight) in the systematic review and meta-analyses.

### 3.2. Study Characteristics

#### 3.2.1. Participant Characteristics

Twenty one (70.00%) studies included women of any BMI at baseline, seven (23.33%) included women with overweight or obesity only, and one (3.33%) included women with obesity only [24]. At baseline, the mean age ranged from 26.12 [25] to 43 years [26], mean BMI from 20.7 [27] to 35.20 kg/m^2^ [28], and mean body weight from 54.10 [25] to 92.98 kg [28].

Fifteen (50.0%) of the interventions started within one year postpartum, three (10.0%) started during pregnancy [29,30,31], one started one year or more postpartum, and the remaining eleven (36.67%) had overlapping or not explicitly stated commencement times (Table 1).

#### 3.2.2. Intervention Characteristics According to TIDieR Checklist

##### Item 1. Name of the Intervention

While about half of the authors generally described their intervention as a lifestyle intervention for women with a history of GDM (*n* = 14), the other studies described their interventions as a group-based lifestyle intervention (*n* = 1) [24], web-based intervention (*n* = 2) [32,33], eHealth lifestyle programme (*n* = 1) [34], phone-based lifestyle intervention (*n* = 1) [35], cultural lifestyle intervention (*n* = 1) [36], individualized early postpartum lifestyle intervention (*n* = 1) [37], text messaging (*n* = 1) [27], clinically based Mediterranean lifestyle intervention (*n* = 1) [38], walking for exercise and nutrition (*n* = 1) [39], translating healthy living messages (*n* = 1) [28], postpartum diabetes mellitus prevention programme/intervention (*n* = 2) [40,41], structured behavioural intervention (*n* = 1) [42], system-based lifestyle intervention (*n* = 1) [29], complex interdisciplinary lifestyle and psychosocial intervention (*n* = 1) [31], blended mobile-based lifestyle intervention (*n* = 1) [43], and early postnatal lifestyle modification programme (*n* = 1) [44].

##### Item 2. Why: The Theory Underpinning the Intervention

Two studies [40,45] were based on social cognitive theory [46], one study [47] cited the Health Belief model [48], one [34] used the Capability, Opportunity, Motivation, Behaviour (COM-B) model [49], and one used the Health Action Process Approach [31] to design the intervention. The rest (*n* = 25) of the studies did not specify a named theory.

##### Items 3 and 4 What: Intervention, Materials, and Procedures Used in the Intervention

Twenty-seven studies used a combination of diet and physical activity, two studies [33,42] used physical activity only, and one used diet only [50]. Twenty-three (76.67%) of the studies mentioned that they provided the participants with tools and resources such as body weight scales, pedometers, printed materials on diet and exercise, websites, logbooks, and dietary and exercise diaries. All of the studies described the procedures that were outlined for the interventions.

##### Item 5. Who Provided the Intervention

Twenty-five (83.33%) of the studies used health professionals such as nurses, dietitians, exercise physiologists, diabetes educators, health educators, nutritionists, physiotherapists, occupational therapists, family medicine specialists, lifestyle coaches, and medical practitioners to deliver the interventions. One (3.33%) study used a trained counsellor to deliver the intervention [42]. The remaining four studies (13.33%) did not explicitly state who delivered the intervention or if the providers were health professionals or not [25,28,33,51].

##### Item 6. How: Intervention Delivery Mode

Two-thirds of the studies (*n* = 20) delivered the intervention to one participant at a time, one (3,33%) used a group mode of delivery, and nine (30.0%) used a combination of individual and group delivery. Twenty-six (86.67%) of the studies used at least one of the following technologies for intervention delivery: phone (*n* = 18), website (*n* = 5), video (*n* = 1) [32], or mobile applications (*n* = 2) [43,52]. Nine interventions were conducted face-to-face; eight were delivered electronically via websites, telephone calls, and text messages or using mobile applications; and thirteen were delivered both face-to-face and electronically.

##### Item 7. Where: Location of the Intervention

Ten of the studies (33.33%) required the lifestyle modifications to be carried out at home, nine (30.0%) at a centre (hospital, clinic, or health centre), seven (23.33%) at both home or a centre, and four (13.33%) at home and in a community (e.g., local mall).

##### Item 8. When and How Much: Session and Duration

The intervention duration ranged from 10 weeks [38,52] to 36 months [53], with a median of 12 (IQR = 9) months. The number of sessions ranged from the equivalent of 1 in-person session [52] to 53 in-person sessions [28], with a median of 11.0 (IQR = 10.57) sessions.

##### Items 9 and 10. Tailoring (Individualized Intervention) and Modification

Twenty-four (80.0%) of the studies tailored the interventions to the characteristics of the study participants, such as providing individualized dietary and physical activity goals. However, none of the studies reported any modification at the intervention level once implementation had started.

##### Items 11 and 12. How Well (Planned and Actual)

The attrition rate of the studies ranged from 3.5 [50] to 54.2% [29], with a mean of 20.50 (±12.5)%. Only one [40] study had high fidelity, eight had medium fidelity, and twenty had low fidelity according to the definition in Supplementary Table S2 (Table 1 and Table S2).

### 3.3. Intervention Characteristics (High-Income vs. Middle-Income Countries)

A comparison of the intervention characteristics between studies conducted in high-income countries and middle-income countries indicated that studies from high-income countries tended to be based on behaviour change theories (12/20 vs. 1/10), with high fidelity (5/20 vs. 0/10), and they used technologies (16/20 vs. 6/10) and had high number of sessions (7/20 vs. 1/10) (Table S8).

### 3.4. Risk of Bias Assessment

Five studies had a low overall risk of bias [26,30,44,45,54], eight had some concerns [25,31,33,43,47,52,55,56], and seventeen had a high risk of bias. The primary domain that contributed to the high risk of bias was deviations from intended interventions (*n* = 14), followed by missing outcome data (*n* = 3). More than two-thirds (21/30) of the studies reported using intention-to-treat analysis. None of the RCTs had a high risk of bias due to the selection of the reported result. Egger’s tests for studies that reported on T2DM and body weight were *p* = 0.035 and *p* = 0.8940, respectively (Figure S1).

**Table 1:** Characteristics of the included studies.

Study; Country	Setting; Tailored	Sample Size; BMI Category at Baseline	Materials Provided: Type: Diet or Physical Activity ^a^	Theory-Based Intervention; Intervention Provider	Delivery Mode; Technology Use: Phone/Web	Intervention Location; Fidelity	Intervention Duration; Number of Sessions ^b^	Intervention Commencement Time
Nicklas 2014 [32] USA	Hospital Yes	75 With overweight or obesity	Yes Combined	Yes Registered dietitian	Individual Website and phone	Home Medium	12 months Medium (7–12)	6 weeks postpartum
McManus 2018 [28] Canada	Tertiary centres No	178 With overweight or obesity	Yes Combined	Yes Study coordinator	Individual and group Phone and e-mail	Local mall and home Low	12 months High (>12)	<3 months postpartum
Peacock 2015 [39] Australia	Tertiary maternity hospital Yes	31 With overweight or obesity	Yes Combined	Yes Accredited practicing dietitians	Individual and group Phone	Home and hospital Medium	3 months Low (1–6.9)	6 months to 2 years postpartum
Ferrara 2016 [30] USA	Perinatal Centre Yes	2280 With overweight or obesity	Yes Combined	Yes Lifestyle coach, a registered dietitian	Individual Phone	Home High	4.5 months (from 6 weeks to 6 months) Low (1–6.9)	During pregnancy
McIntyre 2012 [45] Australia	Homes Yes	28 Unspecified	Yes Combined	Yes Exercise physiologist	Individual Phone	Home Medium	12 weeks Low (1–6.9)	<6 weeks postpartum
O’Reilly 2016 [40] Australia	Clinics Yes	573 Unspecified	Yes Combined	Yes Specially trained healthcare professionals	Individual and group Phone	Home and community High	12 months Medium (7–12)	3 months postpartum
Reinhardt 2012 [35] Australia	Local Area Health diabetes services No	38 Unspecified	Yes Combined	No Diabetes educators	Individual Phone	Home High	6 months High (>12)	6 weeks to 6 months postpartum
Perez-Ferre 2015 [38] Spain	Hospital Yes	237 Unspecified	No Combined	No Nurse, a registered dietitian, endocrinologist and physiotherapist	Group No	Home and hospital Medium	10 weeks (10 weeks between 3 and 6 months) High (>12)	3–6 months postpartum
Shyam 2013 [47] Malaysia	Hospital Yes	77 Unspecified	Yes Combined	Yes Research nutritionist	Individual E-mail and text message	Home and hospital Medium	1 year Medium (7–12)	2 months or more postpartum
Shek 2014 [53] China	Hospital Yes	450 Unspecified	No Combined	No Dietitian and nurse who had received training in dietetics	Individual No	Hospital Medium	36 months Medium (7–12)	<3 years postpartum
Tandon 2022 [54] Bangladesh, India, and Sri Lank	Hospitals No	1612 Unspecified	Yes Combined	No Counsellors with sociology background, nurses, and nurse auxiliaries	Individual and group Phone	Home and research centre Low	12 months High (>12)	3–18 months postpartum
Zilberman-Kravits 2018 [36] Israel	Clinics No	180 Unspecified	Yes Combined	No Nurse, dietician, and sport instructor	Individual and group No	Clinic Low	2 years Medium (7–12)	3–4 months postpartum
Wein 1999 [50] Australia	Women hospital No	200 Unspecified	No Diet only	No Dietitian	Individual Phone	Home Low	51 months Medium (7–12)	Not explicitly stated
Rollo 2020 [34] Australia	Home/Community Yes	29 With overweight or obesity	Yes Combined	Yes Dietitian and exercise physiologist	Individual Phone/e-mail and video call	Home Low	3 months Medium (7–12)	3–24 months postpartum
Cheung 2011 [42] Australia	Hospitals and clinics No	43 Unspecified	Yes Physical activity	No Trained counsellor	Individual Phone	Home Low	12 months Low (1–6.9)	<4 years after GDM
Cheung 2019 [27] Australia	Public hospitals Yes	60 Unspecified	Yes Combined	Yes Dietitian	Individual Phone	Home High	12 weeks High (>12)	Week 1 to week 38 postpartum
Kim 2012 [33] USA	University health system Yes	49 Unspecified	Yes Physical activity	No Not reported	Individual Phone and e-mail	Home Medium	13 weeks High (>12)	6 weeks to 3 years
Hu 2012 [55] China	Community Yes	1180 Unspecified	Yes Combined	No Dietitian and health educator	Individual Phone	Home and health centre Low	24 months Medium (7–12)	1–5 years postpartum
Holmes 2018 [41] UK (Ireland)	Hospital Yes	60 With overweight or obesity	Yes Combined	Yes Health educator	Individual and group Phone and DVD	Medium	6 months Low (1–6.9)	6 weeks to 6 months postpartum
Lee 2022 [29] Malaysia	Primary care clinics Yes	298 Unspecified	Yes Combined	No Family medicine specialist, medical officer, staff nurse, physiotherapist, and dietitian	Individual No	Clinic Low	2 years Low (1–6.9)	During pregnancy
Man 2021 [26] USA	Clinical centres Yes	350 BMI ≥ 24/(≥22 for Asian)	Yes Combined	Yes Case managers with training in nutrition, exercise, or behaviour modification	Individual and group No	Centres High	~3.2 years High (>12)	Mean of 12 years after GDM
O’Dea 2015 [24] Ireland	Galway University Hospital No	50 With obesity only	No Combined	No Nurses, dietitians, physical activity specialists, and physician	Group No	Centres Low	12 weeks Medium (7–12)	1–3 years postpartum
Geng 2014 [25] China	Hospital Yes	100 With overweight or obesity	Yes Combined	No Not reported	Individual Phone	Home and centre Low	1 year Low (1–6.9)	4–6 weeks postpartum
Yu 2012 [57] China	Hospital No	126 Unspecified	No Combined	No Trained physicians, diabetes health educators, and nutritionists	Individual No	Home Low	24 months NR	6–8 weeks postpartum
Sheng 2012 [51] China	Hospital Yes	130 Unspecified	Yes Combined	No Not reported	Individual No	Home Low	4 months Low (1–6.9)	6–8 weeks postpartum
Liew 2023 [52] Singapore	Research centre Yes	56 Unspecified	Yes Combined	No Team of health coaches from multi-disciplinary domains	Individual Telephone and web	Home Low	8 weeks Low (1–6.9)	History of GDM in last 10 years
Guo 2013 [56] China	Hospital Yes	100 Unspecified	No Combined	No Not reported	Individual Telephone	Hospital Low	1 year Low (1–6.9)	Early postpartum
Quansah 2024 [31]	University Hospital Yes	Unspecified 179	No Combined	Yes Lifestyle coaches	Individual and group Phone	Hospital Low	12 months High (>12)	pregnancy to 1 year postpartum
Tsoi 2024 [44]	Research Center Yes	Unspecified 79	Yes Combined	No Dietitians and exercise instructors	Individual Telephone (call/SMS)	Research centre Low	12 months Medium (7–12)	6–12 weeks postpartum
Minschart 2024 [43]	Diabetes Clinics Yes	Unspecified 167	Yes Combined	Yes Lifestyle coaches	Individual Smartphone application	Diabetes clinics Low	15 months Medium (7–12)	6–16 weeks postpartum

^a^ Number of sessions: 1 individual/group session = 1 session; 1 online/telephone session = 0.5 session; 1 text/email/contact = 0.25 session; ^b^ Combined: Diet + Physical activity.

### 3.5. Meta-Analyses

#### 3.5.1. Overall Intervention Effects

Lifestyle interventions in women with a history of GDM resulted in a 26% T2DM risk reduction (RR = 0.74; 95% CI: 0.59, 0.94) and 0.93 kg reduction in body weight (MD = −0.93; 95% CI: −1.53, −0.32).

#### 3.5.2. Intervention Effect Based on Participant Characteristics

**Figure 2:**
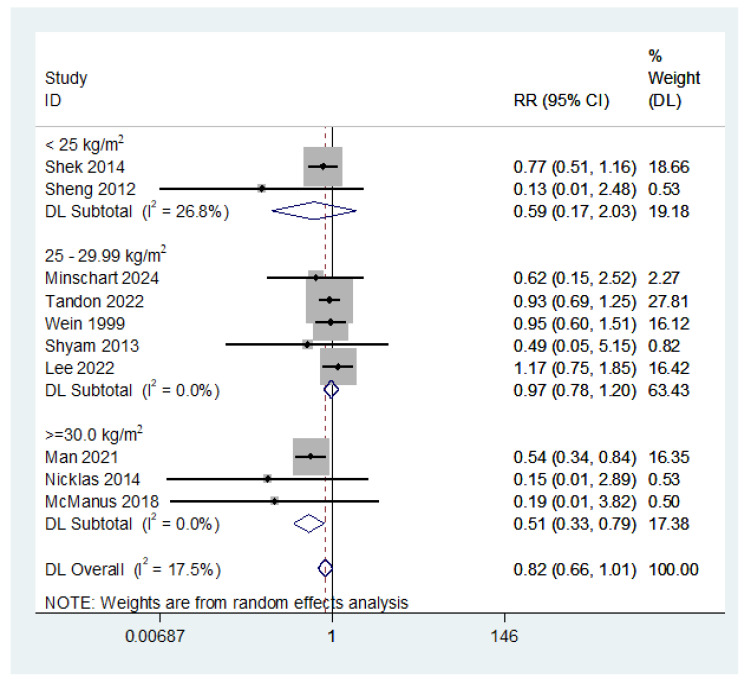
The effect of lifestyle intervention in women with a history of gestational diabetes on type 2 diabetes based on the baseline mean body mass index (subgroup differences *p*-value = 0.028) (studies which did not report the baseline BMI were excluded) [26,28,29,32,43,47,50,51,53,54].

The subgroup analysis of T2DM showed that lifestyle interventions only effectively reduced T2DM in studies in which the participants’ baseline mean BMI was 30 kg/m^2^ or above (RR = 0.51; 95% CI: 0.33, 0.79) but not in those which included participants with a mean BMI of less than 30 kg/m^2^ (Figure 2).

Similarly, lifestyle interventions only effectively reduced the body weight in studies in which the participants’ baseline mean BMI was 25 kg/m^2^ or above, with the greatest effect size seen in those in which the participants’ baseline mean BMI was 30 kg/m^2^ or more (MD = −2.03; 95% CI: −3.05, −1.01) (Figure S2.14).

##### Intervention Effect Based on General Intervention Characteristics and TIDieR Checklist

**Figure 3:**
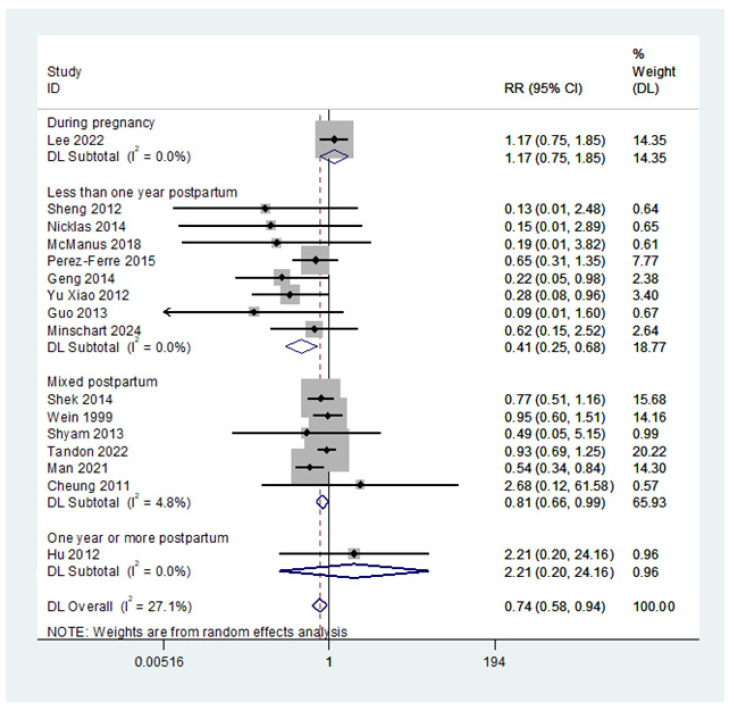
The effect of lifestyle interventions in women with a history of gestational diabetes on type 2 diabetes based on the intervention commencement time (subgroup differences *p*-value = 0.017). Studies that described the time as less than *x* years or at least *y* months or a mean of *z* years were categorized as mixed postpartum [25,26,28,29,32,38,42,43,47,50,51,53,54,55,56,57].

Trials that started within one year postpartum showed a more significant reduction in the incidence of T2DM (RR = 0.41; 95% CI: 0.25, 0.68) compared with trials commencing beyond this period (Figure 3).

**Figure 4:**
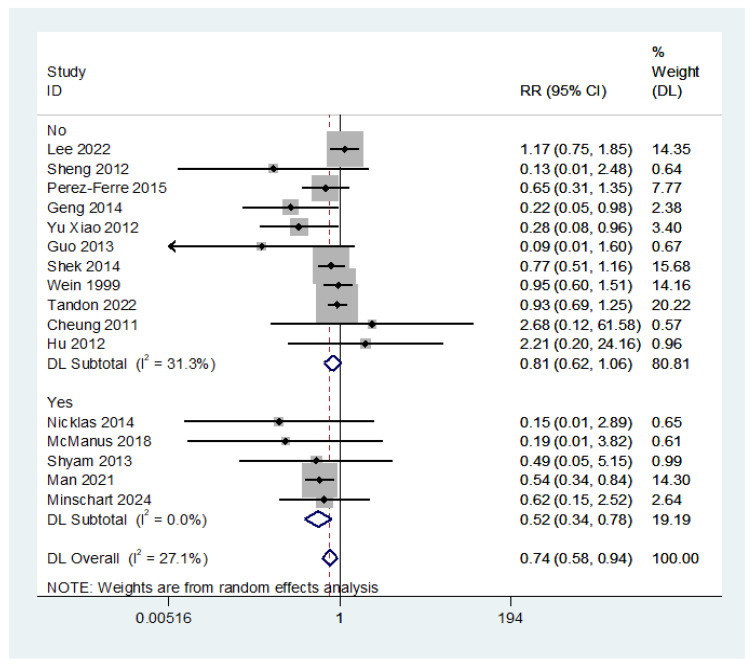
The effect of lifestyle intervention in women with a history of gestational diabetes on type 2 diabetes (theory-based vs. non-theory-based interventions) (subgroup differences *p*-value = **0.028**). For body weight, interventions that were delivered electronically via websites, telephone, or text messages or used both distant and face-to-face approaches resulted in a significantly greater weight reduction than those that were delivered face-to-face only. The effect size was the highest for web-based interventions (MD = −2.45; 95% CI: −3.50, −1.41) and the lowest for face-to-face interventions (MD = 0.97; 95% CI: 0.39, 1.54) (Figure S2.19) [25,26,28,29,32,38,42,43,47,50,51,53,54,55,56,57].

The subgroup analyses based on TIDieR characteristics showed that only studies that were based on behaviour change theories showed a significant reduction in T2DM, with a risk reduction of 48% (RR = 0.52; 95% CI: 0.34, 0.78) (Figure 4).

## 4. Discussion

This systematic review and meta-analysis examined the effect of lifestyle interventions in women with a history of GDM on T2DM incidence and body weight, focusing on the effect of the participant and intervention characteristics on the effect size. Overall, lifestyle interventions in women with a history of GDM showed beneficial effects in reducing the incidence of T2DM and in reducing body weight, which is consistent with the most recent systematic review and meta-analysis [12]. The subgroup analyses of the intervention effect based on the participant and intervention characteristics revealed that trials that started within one year postpartum showed a more beneficial effect in reducing T2DM. Moreover, the beneficial effect was only seen in studies with participants with mean BMIs of 30 kg/m^2^ or above at baseline and in those based on behaviour change theories. For body weight, only studies with participants with a mean BMI of 25 kg/m^2^ or more at baseline and interventions involving electronic or mobile technology via websites, phone calls, or text messaging resulted in more significant body weight reductions. No other association was seen between participant or intervention characteristics and T2DM risk or body weight reduction.

The subgroup analysis based on the participants characteristics found that the T2DM risk reduction was only significant in studies with a baseline mean BMI of 30 kg/m^2^ or above. The body weight was also significantly reduced in studies that included women with a baseline mean BMI of 25 kg/m^2^ or above, suggesting greater intervention effectiveness in individuals with overweight or obesity. BMI and T2DM had a strong positive linear association, as shown in a systematic review that included 2.3 million individuals, suggesting that individuals with overweight or obesity are at higher risk of developing T2DM compared to individuals with healthy weights [58]. Obesity is a stronger predictor of T2DM than genetic predisposition [59]. A greater intervention effectiveness in this group may reflect the greater potential for weight reduction and T2DM risk reduction through lifestyle interventions. While this implies that those with a higher body weight should be targeted for lifestyle interventions for T2DM prevention, caution is needed with intervention messaging to avoid exacerbating weight stigma in this population [60].

Past reviews suggested greater effectiveness in interventions starting at an earlier postpartum stage (e.g., within six months to three years), although they did not find a significant subgroup effect for the intervention commencement postpartum age [10,11]. In the current study, our meta-analysis showed that interventions that started within one year postpartum had a significantly greater T2DM risk reduction effect (59%) compared to other interventions commencing at other times. The risk of conversion to T2DM after GDM is highest between 3 and 6 years postpartum, according to recent systematic review of 2,626,905 participants [61]. Hence, starting lifestyle interventions postpartum before this critical period may prevent or delay the pathophysiological changes that lead to the development of T2DM in women with a history of GDM.

We also found that behaviour-change-theory-based interventions resulted in a more significant reduction in T2DM than interventions that were not underpinned by a behaviour change theory. This finding supports a recent systematic review by Rhoon et al. from 2020, in which more behaviour change techniques were used in effective interventions than ineffective ones [62]. Murimi et al., 2016, also found that using theories in interventions enhances the success of nutrition education interventions [63]. Together, current and past evidence supports the need for diabetes prevention programmes with a clear underpinning behaviour change theory for better effect. As most of the behavioural-change-theory-based studies included in this review are from high-income countries, it could be one of the contributors to the disparities in the diabetes burden between high-income and LMICs. Therefore, researchers and diabetes prevention programmes outside high-income countries, where more than three out of four adults with diabetes live, need to pay special attention to the need for theory-based interventions to reduce inequity in the prevalence of the disease [1].

Our current meta-analysis found that interventions that were delivered via electronic or mobile technologies such as websites, phone calls, or messaging or that used both technologies and face-to-face approaches achieve more reduced body weights than those without these technologies. While interventions in the general population effectively reduced weight in both in-person and virtual approaches [64,65], it appears that interventions that are delivered using internet or phone technologies are more effective in postpartum women with a history of GDM [66]. This could be due to electronic or mobile health delivery addressing some of the reported barriers that are experienced by women postpartum, such as a lack of social support and the need for childcare [67,68,69]. Other benefits of the electronically delivered interventions include access for women who live in rural and remote locations, those without access to transportation, and other circumstances, such as the restricted movement imposed during the COVID-19 pandemic [70]. However, caution is needed in interpreting this finding. More than 80% of the technology-delivered interventions also utilized theory-based approaches, a variable that was found to be important in determining intervention effectiveness in this study. In addition, most of the studies that used technologies are from high-income countries. Greater understanding of the benefit of virtual care applications, particularly in low- and middle-income countries and for women who experience systemic disadvantages (e.g., by location, socioeconomic, or lack of access to the internet), will be needed.

### Strength and Limitations

This study has several limitations that should be considered. First, most of the trials included have a high risk of bias, mainly due to the domain of deviation from the intended intervention, as blinding participants or intervention providers is impossible in diabetes prevention programmes. Second, there is a publication bias, particularly for the studies that reported on T2DM, such that those with negative findings, were less likely to be published. Third, most studies did not report according to the TIDieR checklist, and the data coding may be subjected to the reviewers’ interpretation. Moreover, as we have not limited the search by date, readers need to note the changes in the GDM diagnostic criteria, which have evolved over time [71]. The main strength of our systematic review and meta-analysis is the use of the TIDieR checklist, providing a framework for the optimization of diabetes prevention programmes. Moreover, this study is inclusive of global publications, with no language restrictions.

## 5. Conclusions

This systematic review and meta-analyses found that lifestyle interventions in women with a history of GDM have shown beneficial effects in reducing the risk of T2DM and body weight. The beneficial effect is more significant in women with obesity, in interventions started within one year postpartum, in those underpinned by behaviour change theories, and in interventions that were delivered with electronic or mobile technology. Interventions targeting women with a history of GDM should start within one year postpartum, be underpinned by behavioural change theory, and ensure appropriate messaging to engage those with overweight or obesity. Further studies are needed to determine the optimum delivery methods in lower- and middle-income countries, which have the greatest burden of diabetes globally.

## Data Availability

The protocol was registered in the International Prospective Register of Systematic Reviews (PROSPERO): registration ID CRD42022314231.

## Supplementary Materials

The following supporting information can be downloaded at https://www.mdpi.com/article/10.3390/nu16244413/s1, Table S1. Search strategy (MEDLINE); Table S2. Definition of some TIDieR components; Table S3. Risk of bias assessment; Table S4. Subgroup analyses of the effect of lifestyle intervention in women with a history of GDM on incidence of T2DM by participants baseline anthropometries and intervention characteristics (TIDieR); Table S5. Subgroup analyses of the effect of lifestyle intervention in women with a history of GDM on body weight by participants baseline anthropometries and intervention characteristics (TIDieR); Table S6. Comparison of intervention characteristics of studies form high-income countries and middle-income countries by TIDieR characteristics and effectiveness in reducing the risk of T2DM; Table S7. Comparison of intervention characteristics of studies form high-income countries and middle-income countries by TIDieR characteristics and effectiveness in reducing body weight; Table S8. Comparison of intervention characteristics of studies form high-income countries and middle-income countries; Figure S1. Funnel plots for publication bias; Figure S2. Forest plots.
