# Supplementary material for: A Systematic Review and Meta-Analysis of Type 2 Diabetes Prevention Through Lifestyle Interventions in Women with a History of Gestational Diabetes—A Summary of Participant and Intervention Characteristics
Source: Nutrients. 2024 Dec 23;16(24):4413. doi: 10.3390/nu16244413 (PMC11679762; doi:10.3390/nu16244413)
Supplement: Supplementary file 1 [file nutrients-16-04413-s001.zip › nutrients-3378190-supplementary.pdf]

Supplementary file

Table S1. Search strategy (MEDLINE)

Table S2. Definition of some TIDieR components

Table S3. Risk of bias assessment

Table S4. Subgroup analyses of the effect of lifestyle intervention in women with a history of GDM on incidence of T2DM by participants baseline anthropometries and intervention characteristics (TIDieR)

Table S5. Subgroup analyses of the effect of lifestyle intervention in women with a history of GDM on body weight by participants baseline anthropometries and intervention characteristics (TIDieR)

Table S6. Comparison of intervention characteristics of studies form high-income countries and middle-income countries by TIDieR characteristics and effectiveness in reducing the risk of T2DM.

Table S7. Comparison of intervention characteristics of studies form high-income countries and middle-income countries by TIDieR characteristics and effectiveness in reducing body weight

Table S8. Comparison of intervention characteristics of studies form high-income countries and middle-income countries

Figure S1. Funnel plots for publication bias

Figure S2. Forest plots

Supplementary Table S1. Search Strategy (MEDLINE)

#	**Searches**
1	Diabetes, Gestational/
2	gestational diabetes.mp.
3	gestational diabetes mellitus.mp.
4	GDM.mp.
5	1 or 2 or 3 or 4
6	Diabetes Mellitus, Type 2/
7	Glucose Intolerance/
8	diabetes.mp.
9	diabetes mellitus.mp.
10	type 2 diabetes.mp.
11	type 2 diabetes mellitus.mp.
12	Non insulin dependent diabetes mellitus.mp.
13	NIDDM.mp.
14	impaired fasting glucose.mp.
15	IFG.mp.
16	Impaired glucose tolerance.mp.
17	IGT.mp.
18	maternal complication*.mp.
19	6 or 7 or 8 or 9 or 10 or 11 or 12 or 13 or 14 or 15 or 16 or 17 or 18
20	lifestyle.ab,ti.
21	behavio?r.ab,ti.
22	Diet*.ab,ti.
23	"diet reduce*".ab,ti.
24	"diet restricted*".ab,ti.
25	"nutriti*".ab,ti.
26	"educat*".ab,ti.
27	"exercis*".ab,ti.
28	"physical activit*".ab,ti.
29	20 or 21 or 22 or 23 or 24 or 25 or 26 or 27 or 28
30	"therap*".ab,ti.
31	"intervention*".ab,ti.

32	30 or 31
33	29 and 32
34	5 and 19 and 33

Supplementary Table S2. Definition of some TIDieR components

TIDieR component	Clarification
Theoretical framework	If the researchers mentioned that the study was based on any theory or described the rationale of the intervention. e.g., a named behavior change theory).
Intervention materials	Provision of any physical or informational materials to the study participants. e.g., booklets, lessons, pedometers, mobile applications etc.
Intervention tailoring	Any personalization, individualization, or intervention adaptation at the individual level.
Intervention modification	Any unexpected change or modification at the study level during the study intervention period.
Number of sessions	1 individual/group session = 1 session<br>1 online/telephone session = 0.5 session<br>1 text/email/contact = 0.25 session<br><br>The total number of sessions was categorized as:<br>▪ **Low:** 1 – 6.9 sessions<br>▪ **Medium**: 7 – 12 sessions<br>▪ **High**: more than 12 sessions
Delivery mode	▪ **Individual:** if the intervention was delivered to one participant at a time.<br>▪ **Group:** if the intervention was delivered to more than one participant at a time.
Intervention location	**Hospital/ center/clinic:** if the lifestyle intervention took place at a hospital, health centre or a clinic.<br>**Home or community:** if the lifestyle intervention took place at home or in the community, e.g., web-based interventions.
Intervention provider	**Health professionals**:<br>Dietitians, health educators, neonatal specialists, nurses, family medicine specialists, medical officers, nurses, physiotherapists, exercise physiologists, diabetes educators, research nutritionists, the research team<br>**Non-health professionals**:<br>Counselors (Psychology), peer educators, researchers without named clinical qualifications
Intervention duration	Duration of the intervention measured in weeks or months after active intervention (not including follow-ups)<br>▪ **Short:** < 3 months<br>▪ **Medium**: 3 months to 12 months

	▪ **Long:** more than 12 months
Technology use	If the intervention involved at least one of these: telephone, website, email, or video for the intervention delivery (not including phone contact for recruitment of the participants).
Fidelity	Measures of fidelity: quality assurance processes e.g. session recordings, session checklist, a structured curriculum or manual<br><br>▪ **High:** Two or more measures present<br>▪ **Moderate**: At least one measure present<br>▪ **Low:** No measure present

Some of the definitions are adapted from:

Hoffmann, T.C., et al., *Better reporting of interventions: template for intervention description and replication (TIDieR) checklist and guide.* Bmj, 2014. **348**.

Aziz, Z., et al., *A systematic review of real-world diabetes prevention programs: learnings from the last 15 years.* Implementation science, 2015. **10**(1): p. 1-17.

Lim, S., et al., *A systematic review and meta-analysis of intervention characteristics in postpartum weight management using the TIDieR framework: A summary of evidence to inform implementation.* Obesity Reviews, 2019. **20**(7): p. 1045-1056.

Supplementary Table S3. Risk of bias assessment

a) Risk of bias of the included RCT studies

Study	**Randomization process**	**Deviations from intended interventions**	**Missing outcome data**	**Measurement of the outcome**	**Selection of the reported result**	**Overall Bias**
Cheung 2011	Some concerns	High	High	Some concerns	Some concerns	High
Ferrara 2011	Some concerns	Low	High	Low	Low	High
Cheung 2019	Some concerns	High	Low	Low	Low	High
McIntyre 2012	Low	Low	Low	Low	Low	Low
Nicklas 2014	Low	High	Low	Low	Some concerns	High
Man 2021	Low	Low	Low	Low	Low	Low
Shek 2014	High	High	Low	Low	Some concerns	High
Hu 2012	Low	Low	Low	Low	Some concerns	Some concerns
Perze-Ferre 2015	Some concerns	High	Low	Low	Some concerns	High
O'Reilly 2916	Low	High	Low	Low	Low	High
Reinhardt 2012	Some concerns	High	Low	Some concerns	Some concerns	High
Zilberman K. 2018	High	High	Low	Low	Some concerns	High
Wien 1999	Some concerns	High	Low	Low	Some concerns	High
Rollo 2020	Some concerns	High	High	Low	Some concerns	High
Tandon 2022	Low	Low	Low	Low	Low	Low
Shyams 2013	Low	Low	Low	Low	Some concerns	Some concerns
Peacock 2015	Low	High	High	Low	Some concerns	High
Smith 2014	Some concerns	Low	High	Low	Some concerns	High
Lim 2021	Low	Low	Low	Low	Low	Low
McManus 2018	Some concerns	High	Low	Low	Low	High
Kim 2012	Some concerns	Low	Low	Low	Low	Some concerns
O'Dea 2015	Low	Low	High	Low	Low	High
Sheng 2012	Some concerns	High	Low	Some concerns	Low	High

Yu 2012	Some concerns	High	Low	Some concerns	Low	High
Liew 2023	Some concerns	Low	Low	Low	Some concerns	Some concerns
Guo 2013	Some concerns	Low	Low	Low	Some concerns	Some concerns
Geng 2014	Some concerns	Some concerns	Low	Low	Some concerns	Some concerns
Minschart 2024	Low	Some concerns	Low	Low	Low	Some concerns
Tsoi 2024	Low	Low	Low	Low	Low	Low
Quansah 2024	Low	Some concerns	Low	Low	Low	Some concerns

b) Risk of bias of the included cluster-RCT studies

Study	Randomization process	Identification or recruitment of participants	Deviations from intended interventions	Missing outcome data	Measurement of the outcome	Selection of the reported result	Overall Bias
Holmes 2018	Some concerns	Low	Low	High	Low	Some concerns	High
Ferrara 2016	Low	Low	Low	Low	Low	Low	Low
Lee 2022	Low	Some concerns	High	High	Low	Low	High

Supplementary Table S4. Subgroup analyses of the effect of lifestyle intervention in women with a history of GDM on incidence of T2DM by participants baseline anthropometries and intervention characteristics (TIDieR)[a]

**Analyses**	**Number of studies**	**Number of participants**	**Relative risk (95% CI)**	**I²**	**p-value for subgroup difference**
**Baseline mean age[b]**					0.34
< 35 years	11	3,209	0.65 [0.41, 1.02]	36.3	
≥ 35 years	3	855	0.73 [0.53, 1.01]	36.5	
**Baseline mean BMI[c]**					**0.03**
<25 kg/m²	2	580	0.59 [0.17, 2.03]	26.8	
25 – 29.99 kg/m²	5	2,370	0.97 [0.78, 1.20]	0.0	
>=30 kg/m²	3	384	**0.51 [0.33, 0.79]**	0.0	
**Time intervention started[d]**					**0.02**
During pregnancy	1	332	1.17 [0.75, 1.85]	NA	
< 1 year after childbirth	8	1,032	**0.341 [0.25, 0.68]**	0.0	
≥ 1year after childbirth	1	404	2.21 [0.20, 24.16]	NA	
Mixed postpartum	6	2,577	**0.81 [0.66, 0.99]**	4.8	
**Theory based**					**0.03**
No	11	3,707	0.81 [0.62, 1.06]	31.3	
Yes	5	628	**0.52 [0.34, 0.78]**	0.0	
**Intervention type**					0.53
Diet only	1	193	0.95 [0.60, 1.51]	NA	
Physical activity only	1	34	2.68 [0.12, 61.58]	NA	
Combined	14	4,108	0.69 [0.52, 0.91]	32.5	
**Intervention tailored**					0.90
No	5	2,051	0.64 [0.26, 1.53]	37.5	
Yes	11	2,284	0.74 [0.56, 0.96]	30.2	
**Delivery mode**					0.46
Group	1	1,601	0.93 [0.69, 1.25]	NA	
Individual	12	2,188	0.74 [0.52, 1.05]	28.5	
Individual & group	3	546	0.55 [0.38, 0.94]	0.0	
**In-person vs. Electronically delivered[y]**					0.54
In-person and distantly	10	2,899	0.73[0.50, 1.06]	22.7	
In-person only	5	1361	0.74 [0.52, 1.06]	46.0	
Web-based	1	75	0.15 [0.01, 2.89]	NA	
**Intervention location**					0.85
Home & hospital /centre	5	2,419	0.77 [0.51, 1.17]	17.1	
Home or community	6	655	0.51 [0.22, 1.18]	32.6	
Hospital/ centre/clinic	5	1,261	0.74 [0.47, 1.18]	50.1	
**Intervention duration**					0.66
Short (<3months)	1	237	0.65 [0.31, 1.35]	NA	
Medium (3 -12 months)	11	2978	0.79 [0.56, 1.10]	12.2	
Long (>12 months)	4	1,120	0.71 [0.45, 1.11]	64.1	
**Sessions[g]**					0.56
Low (1- 6.9 sessions)	4	596	0.60 [0.18, 2.05]	55.0	

Medium (7-12 sessions)	6	1,366	0.83 [0.61, 1.11]	0.0	
High (>12 sessions)	4	2,147	0.70 [0.49, 1.02]	39.6	
**Fidelity**					0.06
Low	11	3,284	0.79 [0.56, 1.12]	37.7	
Medium to high	5	1,051	0.64 [0.48, 0.85]	0.0	
Materials provided					0.65
Yes	11	3,229	0.74 [0.52, 1.05]	31.6	
No	5	1,106	0.71 [0.49, 1.03]	30.4	

[a] Template for Intervention Description and Replication (TIDieR).

[b] Studies that did not report mean age or reported median age excluded.

[c] Studies that did not report mean BMI excluded

[d] < year postpartum: intervention started within one year for all; studies that described the time as less than *x* years or at least *y* months or a mean of *z* years were categorized as not explicitly stated.

[e] Studies with unclear intervention provider excluded

[f] Distantly: websites, emails, telephone, mobile application, text messages, video calls (telephone calls for appointments are not included).

[g] Studies with unclear number of sessions excluded;

BMI: Body mass index.

Supplementary Table S5. Subgroup analyses of the effect of lifestyle intervention in women with a history of GDM on body weight by participants baseline anthropometries and intervention characteristics (TIDieR)[a]

**Analyses**	**Number of studies**	**Number of participants**	**Mean difference, kg (95% CI)**	**I²**	**p-value for subgroup difference**
**Baseline mean age[b]**					0.29
< 35 years	16	4,858	-0.85 [-1.647, -0.06]	82.8	
≥ 35 years	5	659	-1.37 [-1.92, -0.82]	0.0	
**Baseline mean BMI[c]**					**0.02**
<25kg/m²	3	185	0.13 [-1.03, 1.30]	0.0	
25 – 29.99 kg/m²	9	630	**-0.77 [-1.10, -0.44]**	0.0	
>=30kg/m²	8	4,184	**-2.03 [-3.05, -1.01]**	49.45	
**Time intervention started[d]**					0.79
During pregnancy	3	2,116	-0.56 [-1.08, -0.03]	0.0	
< year postpartum	11	1,162	-1.02 [-2.19, 0.16]	0.0	
≥ 1 year postpartum	1	404	-1.19 [-1.87, -0.51]	NA	
Mixed postpartum	8	1,902	-1.00 [-1.90, -0.09]	55.3.	
**Theory based**					0.09
Yes	12	2,696	-1.40 [-2.32, -0.49]	77.1	
No	11	2,888	-0.41 [-1.10, 0.28]	61.3	
**Intervention type**					0.67
Physical activity only	2	80	-0.36 [-3.01, 2.29]	57.7	
Physical activity & diet	21	5,504	-0.96 [-1.61, -0.32]	78.5	

**Intervention tailored**					0.15
No	5	299	0.60 [-1.51, 2.71]	0.0	
Yes	18	5,285	-1.02 [-1.65, -0.40]	80.5	
**Delivery mode**					0.43
Group	1	36	0.76 [-2.76, 4.28]	NA	
Individual	15	3,065	-0.80 [-1.52, -0.08]	78.9	
Combined	6	2,483	-1.46 [-2.73, -0.20]	55.1	
**In-person vs. Electronically delivered**[y]					**< 0.001**
In-person only	4	568	0.97 [0.39, 1.54]	0.0	
In-person and Telephone & text message /web based	11	3,070	**-1.02 [-1.36, -0.69]**	0.0	
Electronically (phone & text messages/ mobile app	5	1,800	**-0.51 [-0.90, -0.12]**	0.0	
Web-based	3	146	**-2.45 [-3.50, -1.40]**	46.9	
**Intervention location**					0.79
Home & hospital /centre	5	2,205	-1.09 [-1.90, -0.72]	29.9	
Home or community	12	2,638	-0.77 [-1.66, 0.11]	87.24	
Hospital/ centre/clinic	6	741	-1.34 [-2.90, 0.22]	25.5	
**Intervention duration**					0.35
Short (<3months)	4	166	-0.40 [-0.98, 0.18]	0.0	
Medium (3 -12 months)	16	4,837	-1.10 [-1.85, -0.34]	83.4	
Long (>12 months)	3	581	-0.96 [-3.44, 1.53]	0.0	
**Sessions**					0.21
Low (1- 6.9 sessions)	9	2,347	-0.51 [-1.48, 0.46]	82.7	
Medium (7-12 sessions)	8	1,231	-1.58 [-2.43, -0.73]	67.3	
High (>12 sessions)	6	2,006	-0.70 [-1.70, 0.29]	0.0	
**Fidelity**					0.09
Low	16	3,241	-0.56 [-1.25, 0.13]	65.6	
Medium to high	7	2,343	-1.75 [-1.53, -0.32]	84.4	

[a] Template for Intervention Description and Replication (TIDieR).

[b] Studies that did not report mean age or reported median age excluded.

[c] Studies that did not report bassline BMI excluded

[d] < year postpartum: intervention started within one year for all; studies that described the time as less than *x* years or at least *y* months or a mean of *z* years were categorized as not explicitly stated.

[e] Studies with unclear intervention provider excluded

[f] Distantly: websites, emails, telephone, mobile application, text messages, video calls (telephone calls for appointments are not included).

BMI: Body mass index.

Supplementary Table S6. Comparison of intervention characteristics of studies form high-income countries and middle-income countries by TIDieR characteristics and effectiveness in reducing the risk of T2DM.

	**High-income countries (k= 7)**		**Middle-income countries (k= 9)**		**Low-income countries (k=0)**
**Intervention characteristics**	**Number of effective studies**	**Number of ineffective**	**Number of effective studies**	**Number of ineffective**	**Number of any studies**
Tailored					
Yes	1	3	2	5	0
No	0	3	0	2	0
Theory based					
Yes	1	3	2	1	0
No	0	3	0	6	0
**Intervention type**					
Diet alone	0	1	0	0	0
Physical activity alone	0	0	0	0	0
Diet + physical activity	1	5	1	8	0
**Materials provided**					
Yes	1	4	2	5	0
No	0	2	0	2	0
**Intervention provider**					
Health professional	1	4	1	6	0
Non-health professional	0	1	0	0	0
Not clearly stated	0	1	1	1	0
**Mode of delivery**					
Group	0	1	0	0	0
Individual	0	4	2	5	0
Group + individual	1	1	0	2	0
**Technology use**					
Yes	0	5	1	3	0
No	1	1	1	4	0
**Intervention location**					
Home	0	3	1	2	0
Hospital/Health centre	1	2	1	3	0
Home and hospital/centre	0	1	0	2	0
**Fidelity**					
High	1		0	0	0
Medium	0	3	0	2	0
Low	0	3	2	5	0
**Intervention duration**					
$\geq$ 6 months	1	6	2	6	0
< 6 months	0	0	1	0	0
**Session**					
High (>12)	1	2	0	1	0
Medium (7-12)	0	2	0	4	0

Low (1 – 6.9)	0	2	1	2	0
Not clearly stated	0		1	0	0
**Intervention commencement time**					
In one year postpartum	1	2	1	4	0
After one year postpartum	1	2	0	2	0
During pregnancy	0	0	0	1	0
Not clearly stated	0	1	0	1	0

Note: k = number of studies

Supplementary Table S7. Comparison of intervention characteristics of studies form high-income countries and middle-income countries by TIDieR characteristics and effectiveness in reducing body weight

	**High-income countries (k= 16)**		**Middle-income countries (k = 7)**		**Low-income countries (k = 0)**
**Intervention characteristics**	**Number of effective studies**	**Number of ineffective**	**Number of effective studies**	**Number of ineffective**	**Number of any studies**
**Tailored**					
Yes	6	5	1	6	0
No	0	5	0	0	0
**Theory based**					
Yes	6	5	0	1	0
No	0	5	1	5	0
**Intervention type**					
Diet alone	0	0	0	0	0
Physical activity alone	0	2	0	0	0
Diet + physical activity	6	8	1	6	0
**Materials provided**					
Yes	6	8	1	6	0
No	0	2	0	0	0
**Intervention provider**					
Health professional	6	7	1	4	0
Non-health professional	0	0	0	0	0
Not clearly stated	0	3	0	2	0
**Mode of delivery**					
Group	0	1	0	0	0
Individual	4	5	1	5	0
Group + individual	2	4	0	1	0
**Technology use**					
Yes	6	9	1	5	0
No	0	1	0	1	0
**Intervention location**					
Home	4	6	0	2	0
Hospital/Health centre	1	4	0	2	0

Home and hospital/centre	1	0	0	1	0
**Fidelity**					
High	2	1	0	0	0
Medium	3	5	0	1	0
Low	1	4	1	5	0
**Intervention duration**					
≥ 6 months	4	6	1	5	0
< 6 months	2	4	0	1	0
**Session**					
High (>12)	0	5	0	1	0
Medium (7-12)	3	2	1	1	0
Low (1 – 6.9)	3	3	0	2	0
Not clearly stated	0	0	0	2	0
**Intervention commencement time**					
In one year postpartum	5	7	0	4	0
After one year postpartum	0	2	0	1	0
During pregnancy	1	0	0	1	0
Not clearly stated	0	1	1	0	0

Note: k = number of studies

Supplementary Table S8. Comparison of intervention characteristics of studies form high-income countries and middle-income countries

**Intervention characteristics**	**High-income country**	**Middle-income country**
**Materials provided**		
Yes	16 (80.0%)	7 (70.0%)
No	4 (20.0%)	3 (30.0%)
**Intervention Type**		
Diet + physical activity	17 (85.0%)	10 (100%)
Diet only	1 (5.0%)	0 (0%)
Physical activity only	2 (10.0%)	0 (0%)
**Behavioural theory-based**		
Yes	12 (60.0%)	1 (11.0%)
No	8 (40.0%)	9 (90.0%)
**Intervention provider**		
Health professional	19 (95%)	7 (70.0%)
Non-health professional	0 (0%)	0 (0%)
Not clearly stated	1 (5.0%)	3 (30.0%)
**Mode of delivery**		
Group	2 (10.0%)	1 (10.0%)
Individual	11 (55.0%)	0 (0%)
Group + individual	7 (35.0%)	9 (90.0%)

**Technology use**		
Yes	16 (80.0%)	6 (60.0%)
No	4 (20.0%)	4 (40.0%)
**Fidelity**		
High	5 (25.0%)	0 (0%)
Medium	6 (30.0%)	2 (20.0%)
Low	9 (45.0%)	8 (70.0)
**Intervention location**		
Home	12 (60.0%)	2 (20.0%)
Hospital/Health centre	6 (30.0%)	4 (40.0%)
Home and hospital/centre	2 (10.0%)	4 (40.0%)
**Intervention duration**		
≥ 6 months	11 (55.0%)	9 (88.9%)
< 6 months	9 (45.0%)	1 (10.0%)
**Intervention commencement time**		
In one year postpartum	12 (60.0%)	7 (70%)
After one year postpartum	5 (25.0%)	2 (20.0%)
During pregnancy	2 (10.0%)	1 (10.0%)
Not clearly stated	1 (5.0%)	0 (0%)
**Session**		
High (>12)	7 (35.0%)	1 (10.0%)
Medium (7-12)	7 (35.0%)	4 (40.0%)
Low (1 - 6.9)	6 (30.0%)	4 (40.0%)
Not clearly stated	0 (0%)	1 (10.0%)

Supplementary Figure S1. Funnel plots for publication bias of studies testing the effect of lifestyle intervention on Type 2 diabetes and body weight

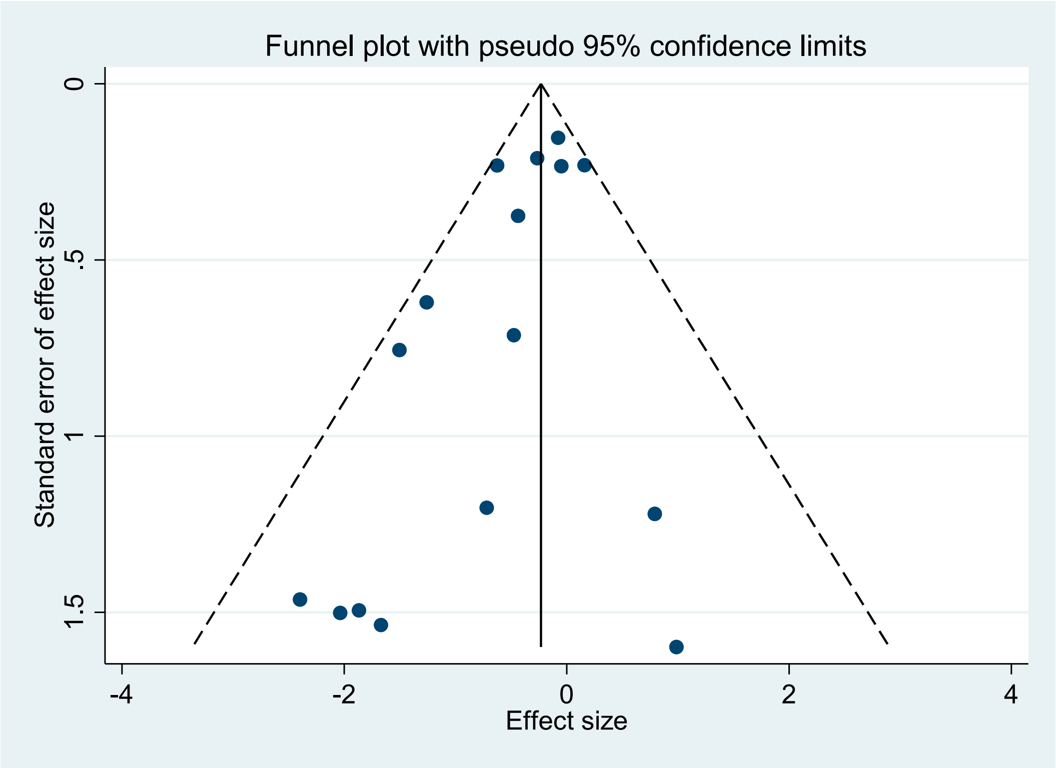

A) Type 2 diabetes: Egger's test P = 0.035

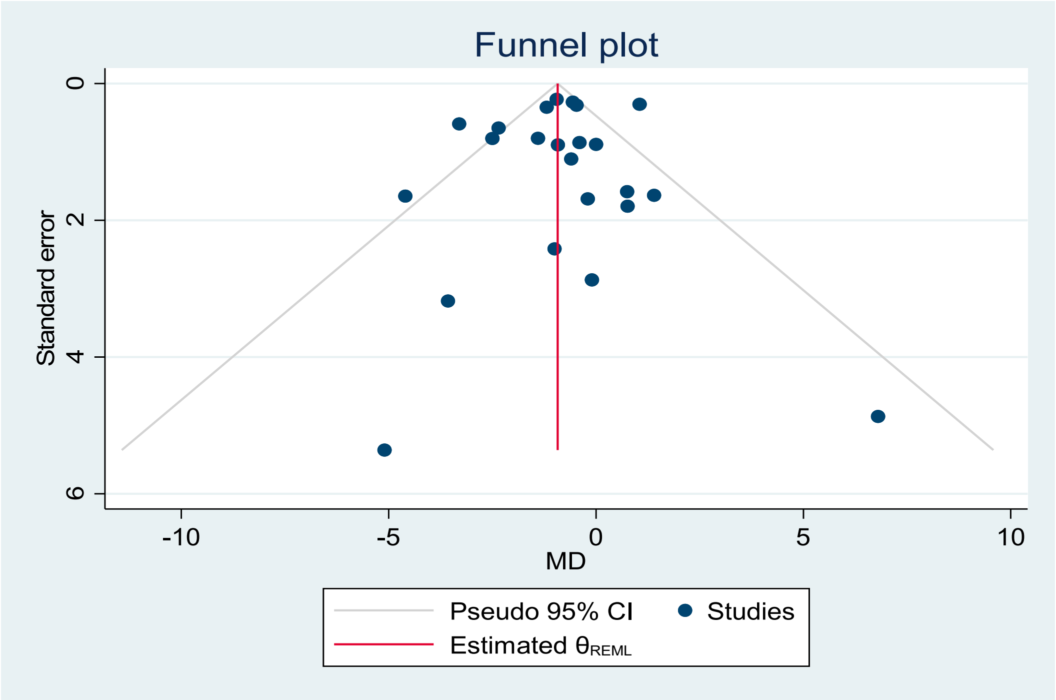

B) Body weight: Egger's test P = 0.8940

Supplementary Figure S2: Forest plots.

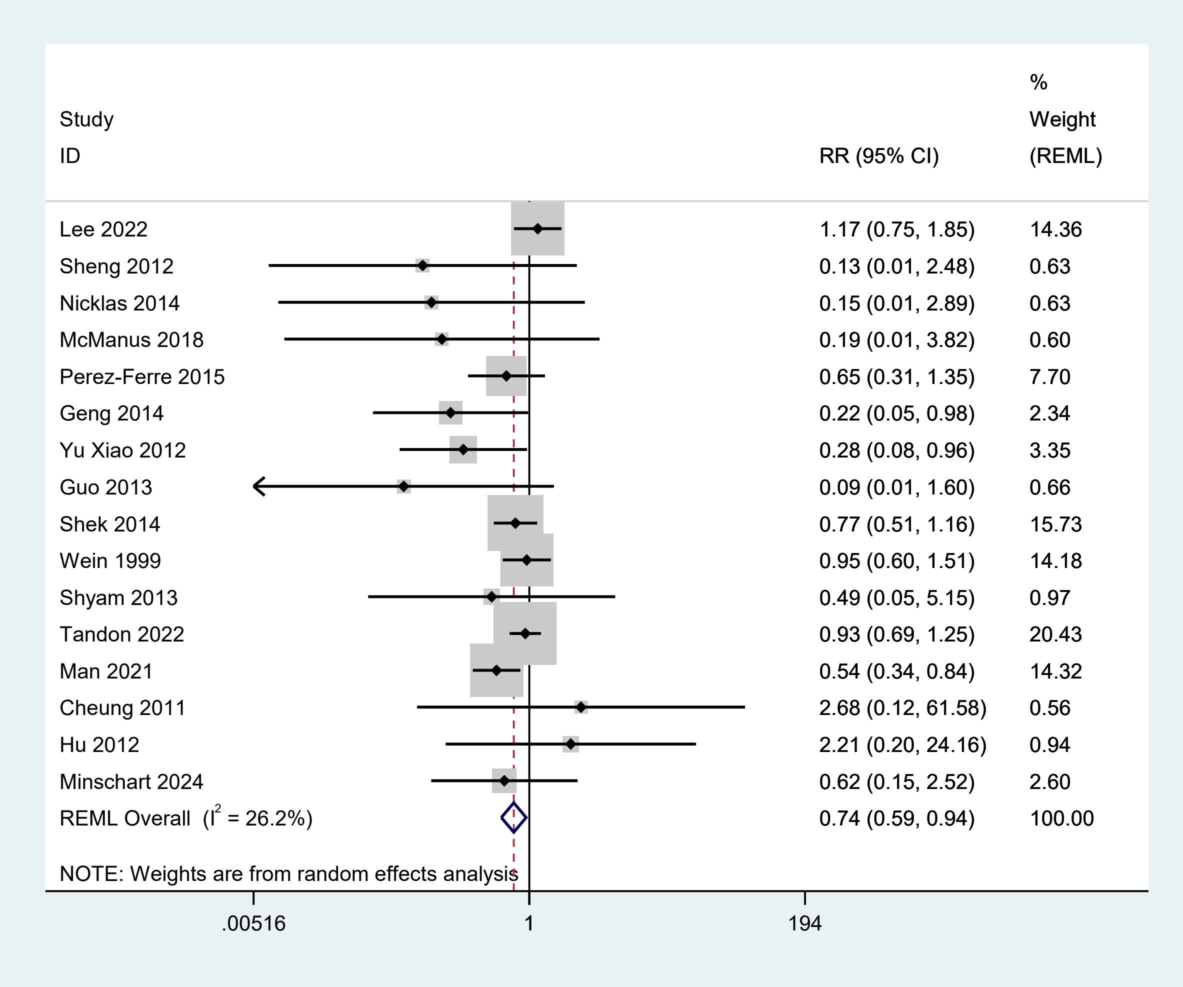

Figure S2.1 The effect of lifestyle intervention in women with a history of gestational diabetes on type 2 diabetes

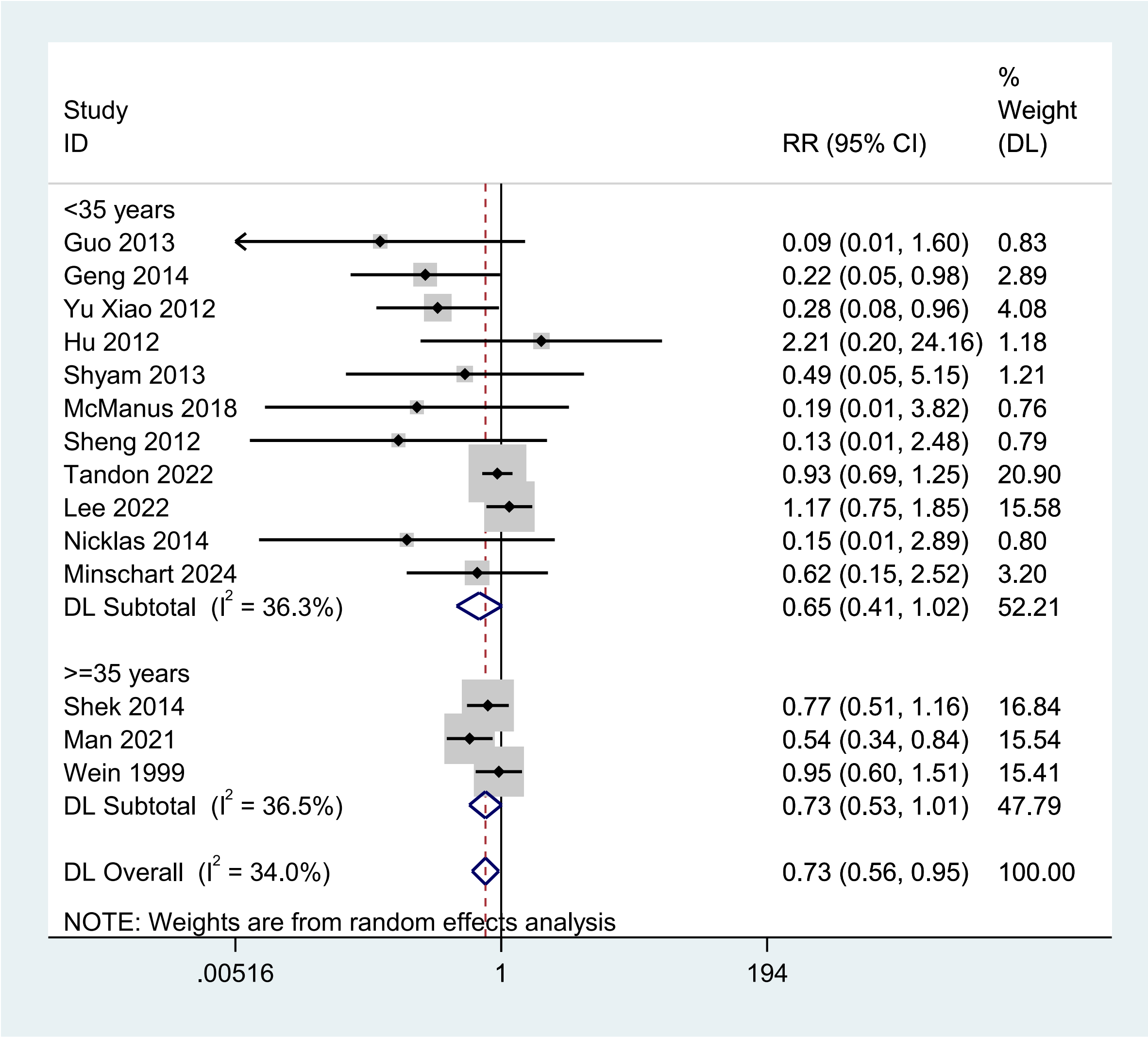

Figure S2.2. The effect of lifestyle intervention in women with a history of gestational diabetes on type 2 diabetes by baseline mean age (Test for subgroup differences: Chi^2=0.83, df=1, p-value=0.3361, H^2=1.00, I^2=0.00)

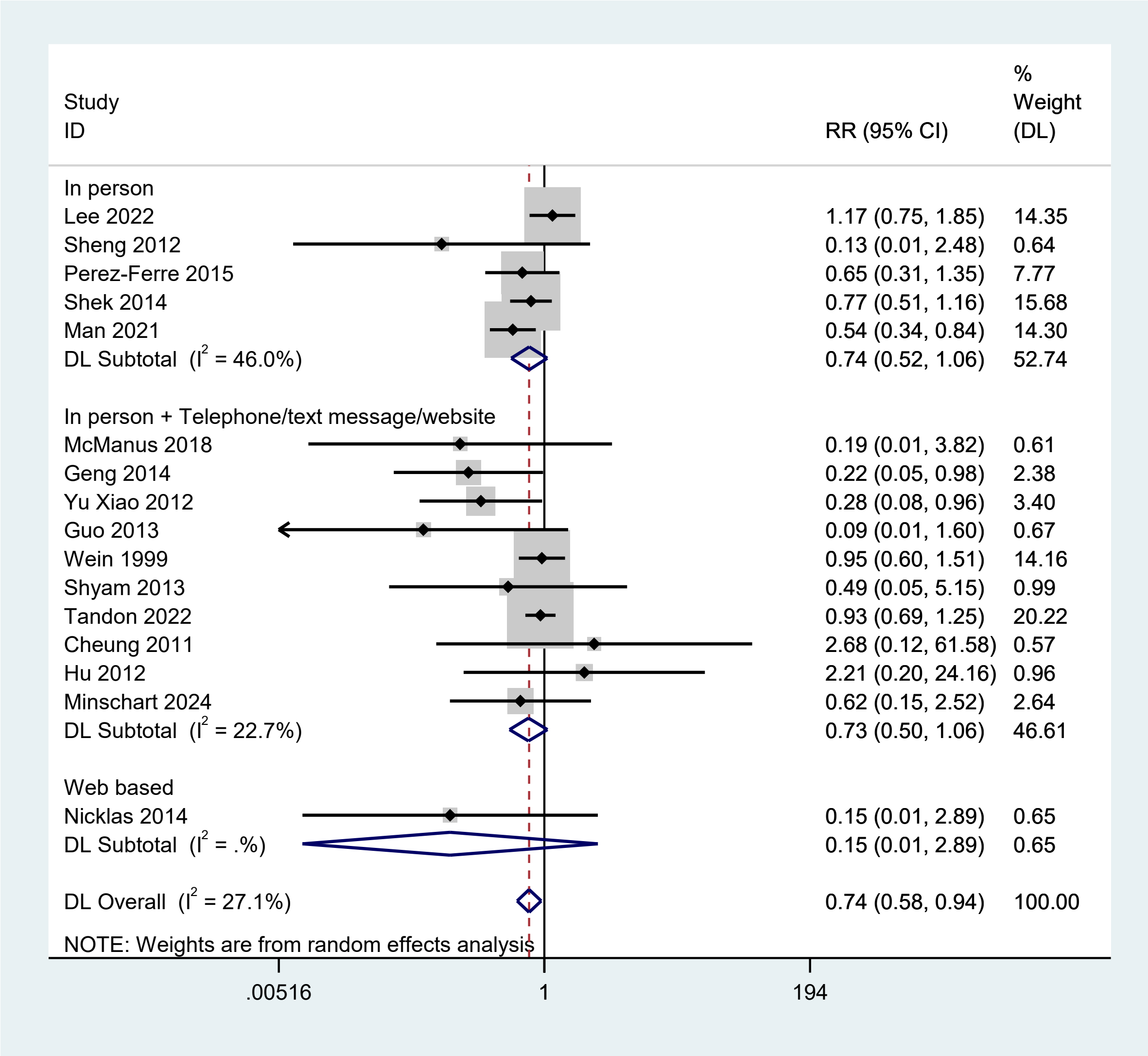

Figure S2.3. The effect of lifestyle intervention in women with a history of gestational diabetes on type 2 diabetes by intervention delivery options (in-person vs distantly delivered) (Test for subgroup differences: Chi^2=1.54, df=2, p-value=0.464, H^2=1.00, I^2=0.00)

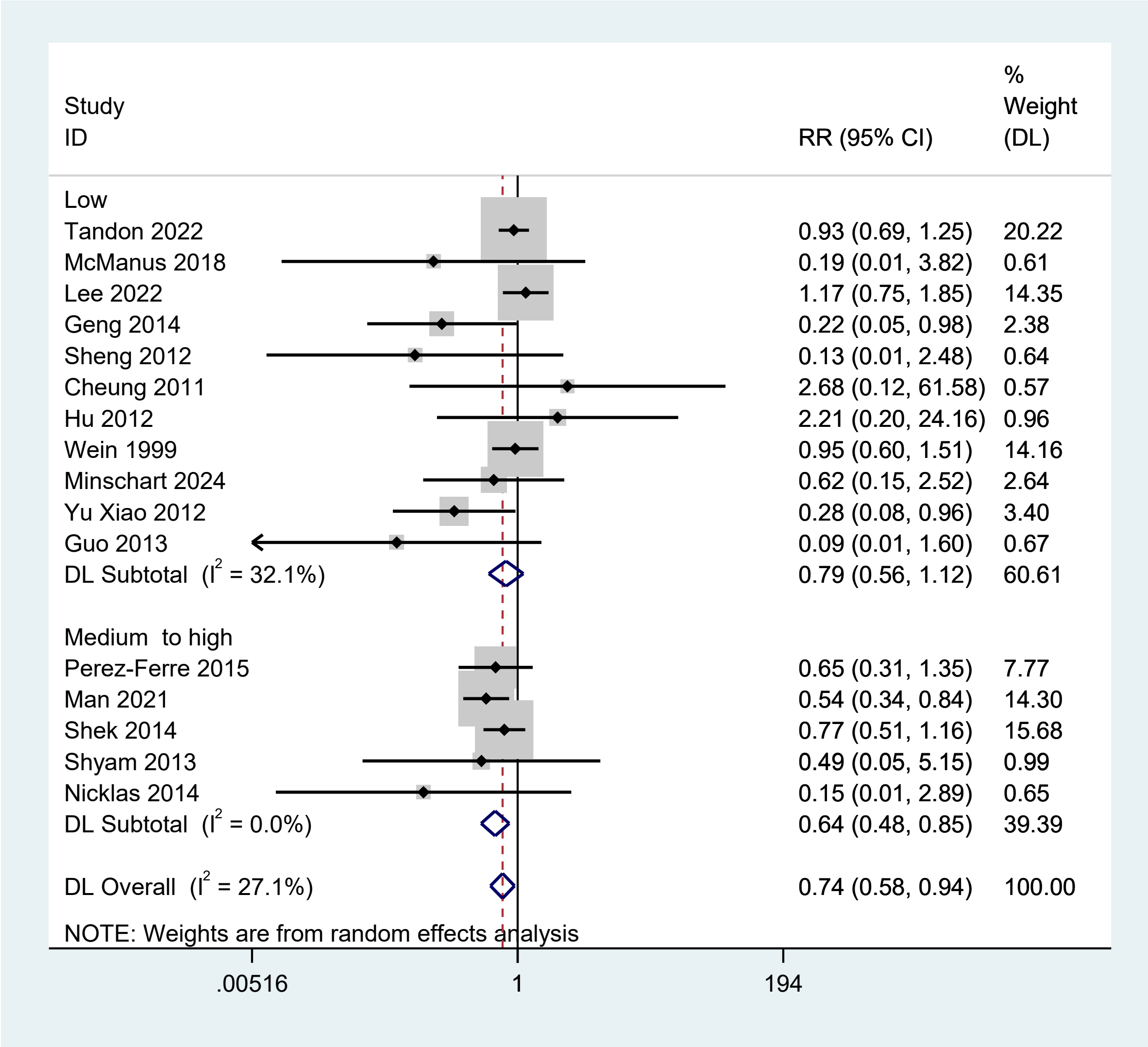

Figure S2.4. The effect of lifestyle intervention in women with a history of gestational diabetes on type 2 diabetes by study fidelity (Test for subgroup differences: Chi^2=3.57, df=1, p-value=0.059, H^2=2.57, I^2=61.02)

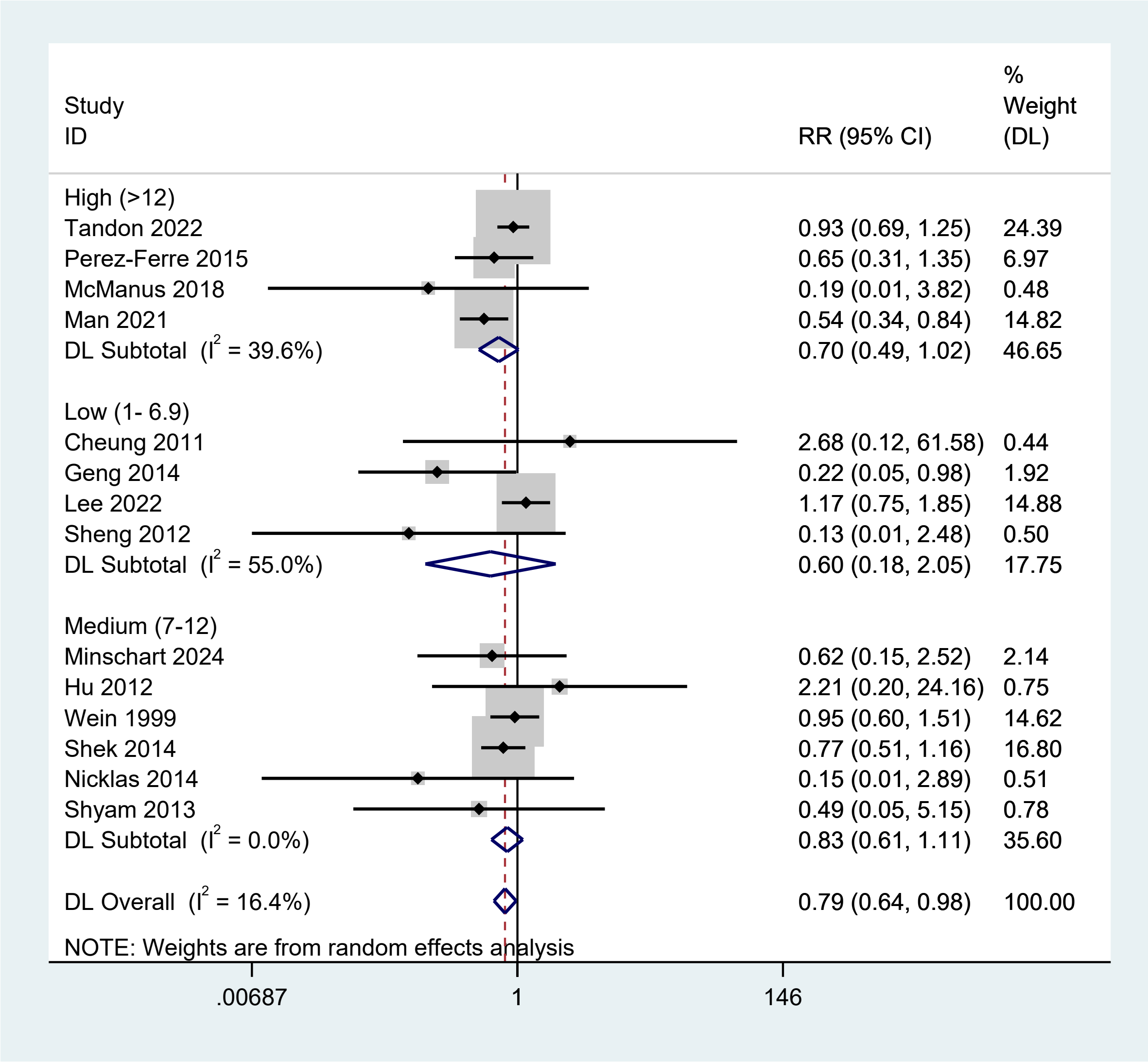

Figure S2.5. The effect of lifestyle intervention in women with a history of gestational diabetes on type 2 diabetes by number of sessions (Test for subgroup differences: Chi^2=1.16, df=2, p-value=0.561, H^2=1.00, I^2=0.00) (Studies with unclear number of sessions are not included).

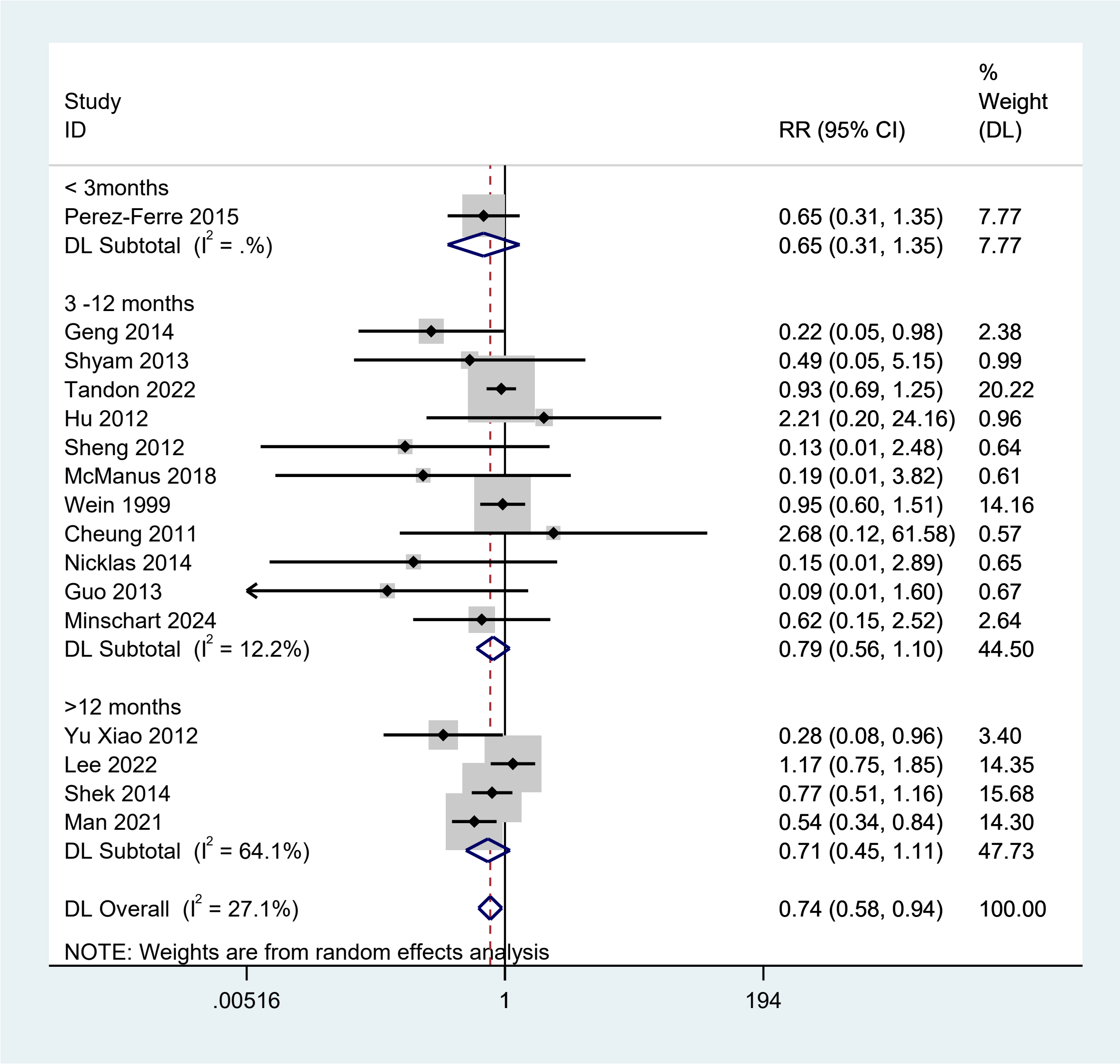

Figure S2.6. The effect of lifestyle intervention in women with a history of gestational diabetes on type 2 diabetes by intervention duration (Test for subgroup differences: Chi^2=0.84, df=2, p-value=0.657, H^2=1.00, I^2=0.00)

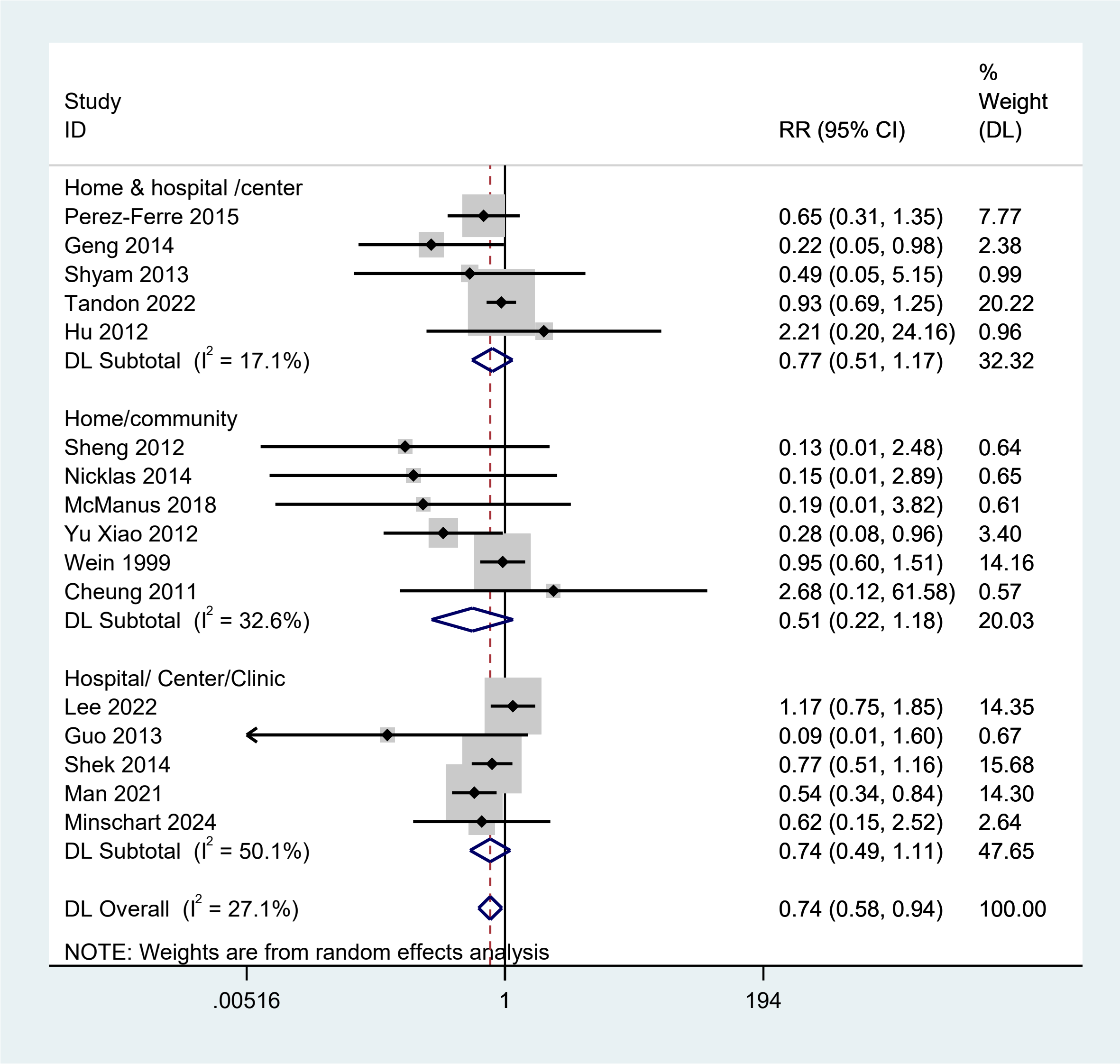

Figure S2.7. The effect of lifestyle intervention in women with a history of gestational diabetes on type 2 diabetes by intervention location (Test for subgroup differences: Chi^2=0.32, df=2, p-value=0.851, H^2=1.00, I^2=0.00)

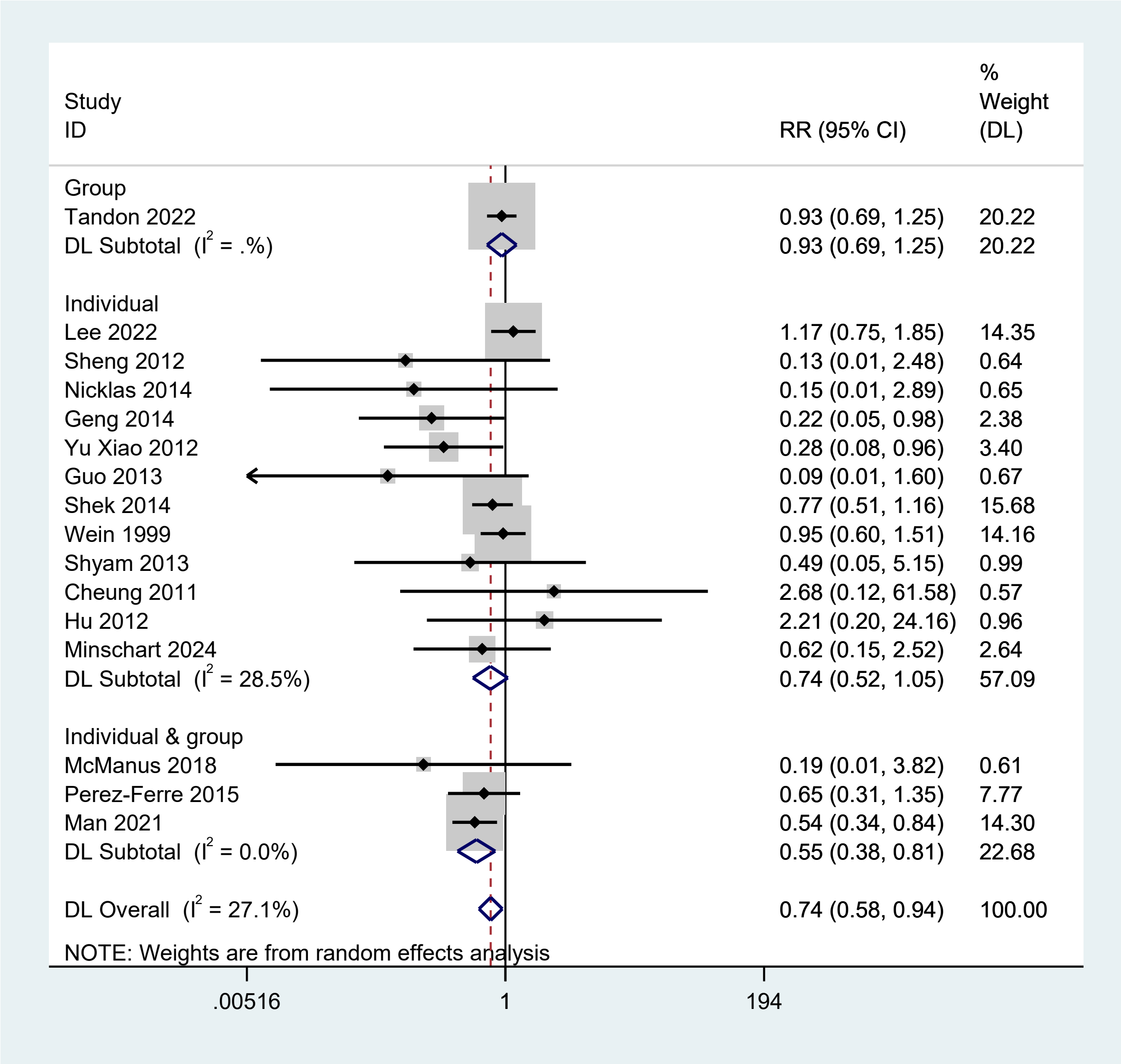

Figure S2.8. The effect of lifestyle intervention in women with a history of gestational diabetes on type 2 diabetes by intervention delivery mode (Test for subgroup differences: Chi^2=4.51, df=2, p-value=0.104, H^2=1.26, I^2=20.56)

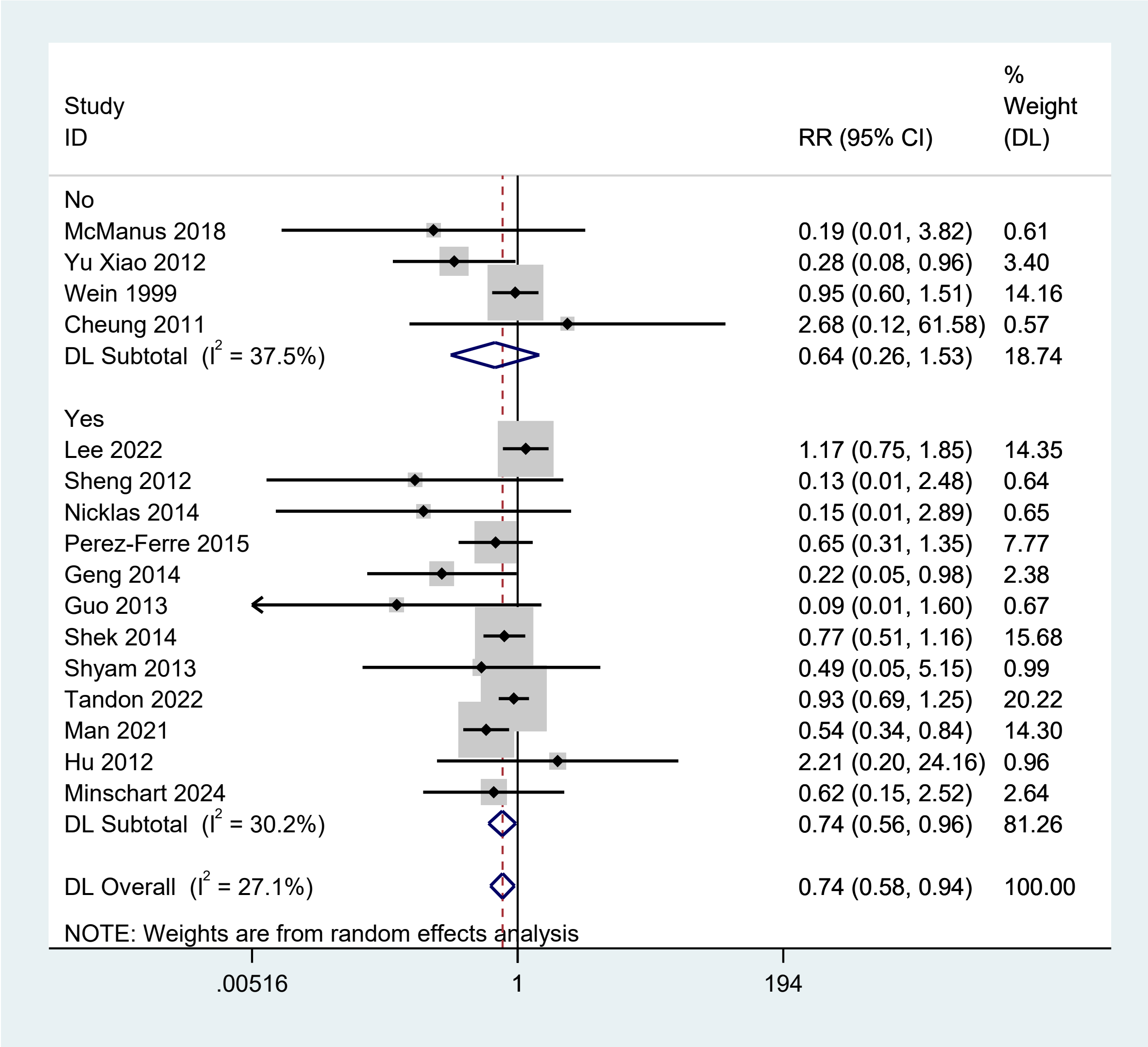

Figure S2.9. The effect of lifestyle intervention in women with a history of gestational diabetes on type 2 diabetes (tailored vs not-tailored interventions) (Test for subgroup differences: Chi^2=0.02, df=1, p-value= =0.899, H^2=1.00, I^2=0.00)

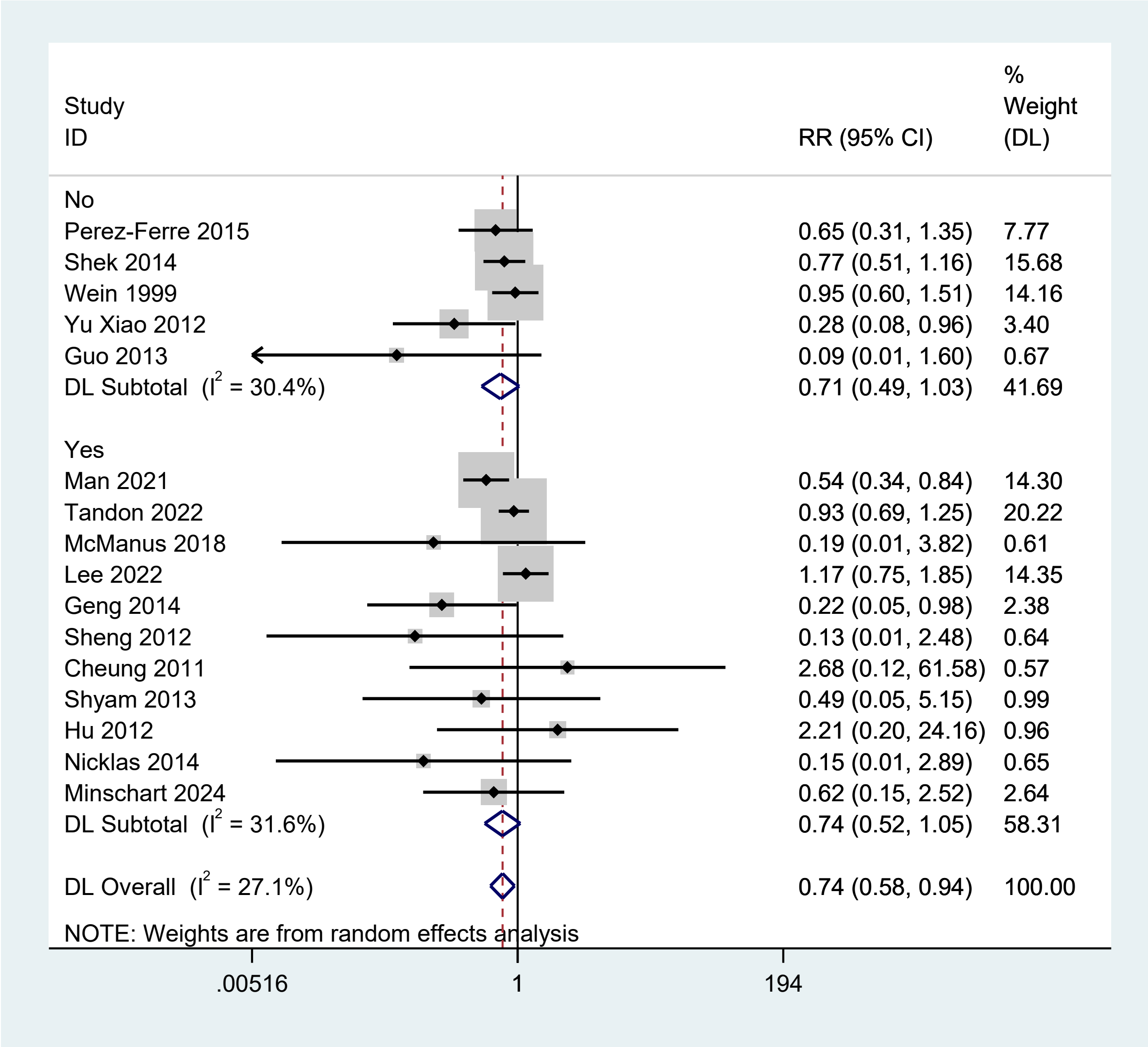

Figure S2.10. The effect of lifestyle intervention in women with a history of gestational diabetes on type 2 diabetes by provision of materials to the participants (Test for subgroup differences: Chi^2=0.20, df=1, p-value=0.652, H^2=1.00, I^2=0.00)

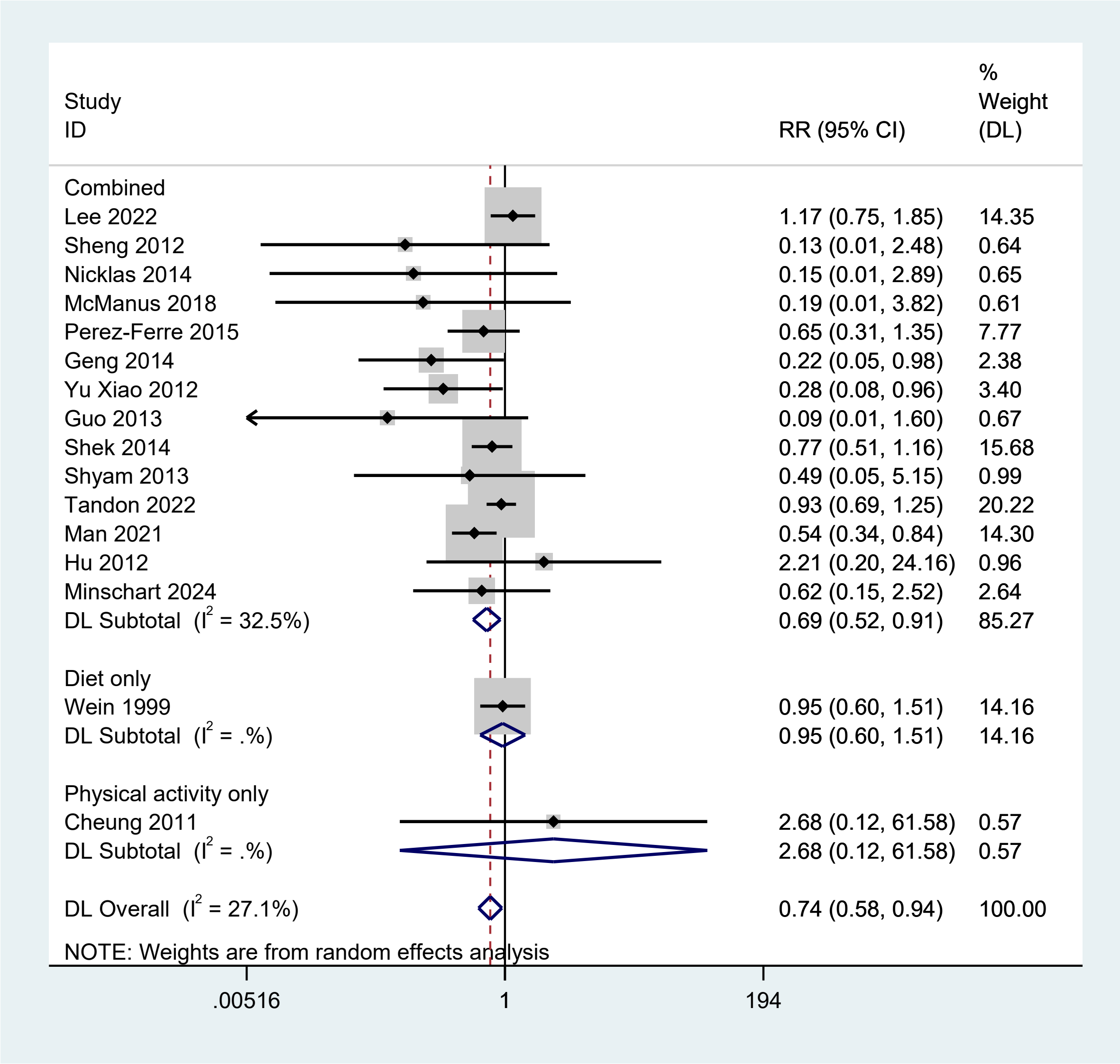

Figure S2.11. The effect of lifestyle intervention in women with a history of gestational diabetes on type 2 diabetes by intervention type (Test for subgroup differences: Chi^2=1.32, df=2, p-value=0.518, H^2=1.00, I^2=0.00)

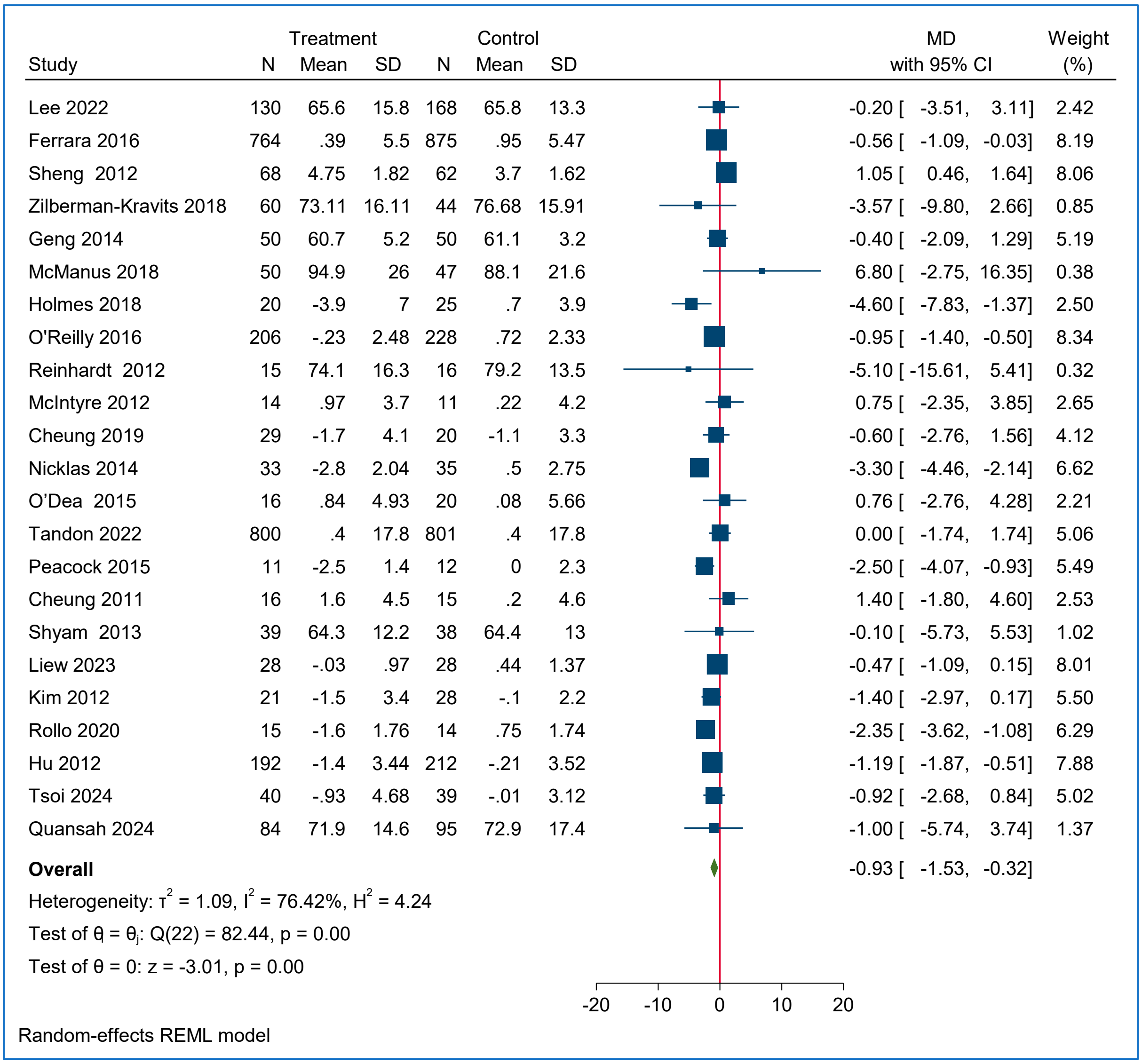

Figure S2.12. The effect of lifestyle intervention in women with a history of gestational diabetes on body weight.

Study	Treatment N	Treatment Mean	Treatment SD	Control N	Control Mean	Control SD	MD with 95% CI	Weight (%)
**< 25 kg/m2**								
Sheng 2012	68	4.75	1.82	62	3.7	1.62	1.05 [0.46, 1.64]	9.18
Cheung 2019	29	-1.7	4.1	20	-1.1	3.3	-0.60 [-2.76, 1.56]	5.05
Liew 2023	28	-.03	.97	28	.44	1.37	-0.47 [-1.09, 0.15]	9.12
Heterogeneity: τ^2 = 0.76, I^2 = 81.42%, H^2 = 5.38							0.13 [-1.03, 1.30]	
Test of $\theta_i = \theta_j$: Q(2) = 12.67, p = 0.00								
Test of θ = 0: z = 0.23, p = 0.82								
**25 - 29.99 kg/m2**								
O'Reilly 2016	206	-.23	2.48	228	.72	2.33	-0.95 [-1.40, -0.50]	9.44
Tandon 2022	800	.4	17.8	801	.4	17.8	0.00 [-1.74, 1.74]	6.09
Shyam 2013	39	64.3	12.2	38	64.4	13	-0.10 [-5.73, 5.53]	1.33
Quansah 2024	84	71.9	14.6	95	72.9	17.4	-1.00 [-5.74, 3.74]	1.77
Lee 2022	130	65.6	15.8	168	65.8	13.3	-0.20 [-3.51, 3.11]	3.06
Ferrara 2016	764	.39	5.5	875	.95	5.47	-0.56 [-1.09, -0.03]	9.30
Reinhardt 2012	15	74.1	16.3	16	79.2	13.5	-5.10 [-15.61, 5.41]	0.42
Tsoi 2024	40	-.93	4.68	39	-.01	3.12	-0.92 [-2.68, 0.84]	6.05
Zilberman-Kravits 2018	60	73.11	16.11	44	76.68	15.91	-3.57 [-9.80, 2.66]	1.11
Heterogeneity: τ^2 = 0.00, I^2 = 0.20%, H^2 = 1.00							-0.77 [-1.10, -0.44]	
Test of $\theta_i = \theta_j$: Q(8) = 3.59, p = 0.89								
Test of θ = 0: z = -4.55, p = 0.00								
**>=30.0 kg/m2**								
McManus 2018	50	94.9	26	47	88.1	21.6	6.80 [-2.75, 16.35]	0.51
Holmes 2018	20	-3.9	7	25	.7	3.9	-4.60 [-7.83, -1.37]	3.16
McIntyre 2012	14	.97	3.7	11	.22	4.2	0.75 [-2.35, 3.85]	3.34
Nicklas 2014	33	-2.8	2.04	35	.5	2.75	-3.30 [-4.46, -2.14]	7.74
Rollo 2020	15	-1.6	1.76	14	.75	1.74	-2.35 [-3.62, -1.08]	7.40
Peacock 2015	11	-2.5	1.4	12	0	2.3	-2.50 [-4.07, -0.93]	6.55
Kim 2012	21	-1.5	3.4	28	-.1	2.2	-1.40 [-2.97, 0.17]	6.56
O'Dea 2015	16	.84	4.93	20	.08	5.66	0.76 [-2.76, 4.28]	2.81
Heterogeneity: τ^2 = 0.92, I^2 = 49.45%, H^2 = 1.98							-2.03 [-3.05, -1.01]	
Test of $\theta_i = \theta_j$: Q(7) = 16.27, p = 0.02								
Test of θ = 0: z = -3.89, p = 0.00								
**Overall**							-1.01 [-1.71, -0.31]	
Heterogeneity: τ^2 = 1.29, I^2 = 78.86%, H^2 = 4.73								
Test of $\theta_i = \theta_j$: Q(19) = 78.48, p = 0.00								
Test of θ = 0: z = -2.84, p = 0.00								
Test of group differences: $Q_b(2)$ = 8.06, p = 0.02								

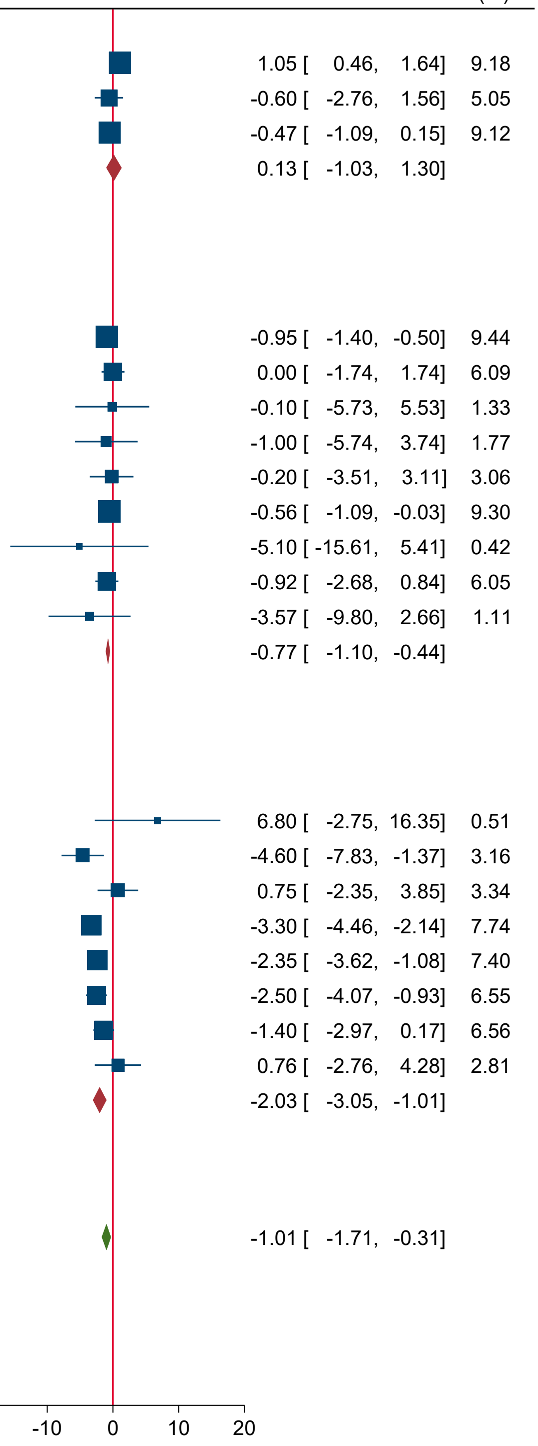

Random-effects REML model

Figure S2.13. The effect of lifestyle intervention in women with a history of gestational diabetes on body weight by baseline mean body mass index

Study	Treatment N	Treatment Mean	Treatment SD	Control N	Control Mean	Control SD	MD with 95% CI	Weight (%)
<35 years								
Geng 2014	50	60.7	5.2	50	61.1	3.2	-0.40 [-2.09, 1.29]	5.44
McManus 2018	50	94.9	26	47	88.1	21.6	6.80 [-2.75, 16.35]	0.40
Holmes 2018	20	-3.9	7	25	.7	3.9	-4.60 [-7.83, -1.37]	2.62
O'Reilly 2016	206	-.23	2.48	228	.72	2.33	-0.95 [-1.40, -0.50]	8.75
Tandon 2022	800	.4	17.8	801	.4	17.8	0.00 [-1.74, 1.74]	5.31
Shyam 2013	39	64.3	12.2	38	64.4	13	-0.10 [-5.73, 5.53]	1.07
Quansah 2024	84	71.9	14.6	95	72.9	17.4	-1.00 [-5.74, 3.74]	1.44
Lee 2022	130	65.6	15.8	168	65.8	13.3	-0.20 [-3.51, 3.11]	2.54
Sheng 2012	68	4.75	1.82	62	3.7	1.62	1.05 [0.46, 1.64]	8.47
Ferrara 2016	764	.39	5.5	875	.95	5.47	-0.56 [-1.09, -0.03]	8.60
Reinhardt 2012	15	74.1	16.3	16	79.2	13.5	-5.10 [-15.61, 5.41]	0.33
McIntyre 2012	14	.97	3.7	11	.22	4.2	0.75 [-2.35, 3.85]	2.78
Cheung 2019	29	-1.7	4.1	20	-1.1	3.3	-0.60 [-2.76, 1.56]	4.33
Nicklas 2014	33	-2.8	2.04	35	.5	2.75	-3.30 [-4.46, -2.14]	6.95
Liew 2023	28	-.03	.97	28	.44	1.37	-0.47 [-1.09, 0.15]	8.40
Rollo 2020	15	-1.6	1.76	14	.75	1.74	-2.35 [-3.62, -1.08]	6.61
Heterogeneity: τ^2 = 1.42, I^2 = 82.87%, H^2 = 5.84							-0.85 [-1.64, -0.06]	
Test of $\theta_i = \theta_j$: Q(15) = 70.46, p = 0.00								
Test of θ = 0: z = -2.10, p = 0.04								
>= 35 years								
Peacock 2015	11	-2.5	1.4	12	0	2.3	-2.50 [-4.07, -0.93]	5.76
Hu 2012	192	-1.4	3.44	212	-.21	3.52	-1.19 [-1.87, -0.51]	8.27
Tsoi 2024	40	-.93	4.68	39	-.01	3.12	-0.92 [-2.68, 0.84]	5.27
Zilberman-Kravits 2018	60	73.11	16.11	44	76.68	15.91	-3.57 [-9.80, 2.66]	0.89
Kim 2012	21	-1.5	3.4	28	-.1	2.2	-1.40 [-2.97, 0.17]	5.77
Heterogeneity: τ^2 = 0.00, I^2 = 0.00%, H^2 = 1.00							-1.37 [-1.92, -0.82]	
Test of $\theta_i = \theta_j$: Q(4) = 2.98, p = 0.56								
Test of θ = 0: z = -4.88, p = 0.00								
**Overall**							-1.03 [-1.65, -0.41]	
Heterogeneity: τ^2 = 1.09, I^2 = 77.88%, H^2 = 4.52								
Test of $\theta_i = \theta_j$: Q(20) = 80.13, p = 0.00								
Test of θ = 0: z = -3.26, p = 0.00								
Test of group differences: $Q_b(1)$ = 1.10, p = 0.29								

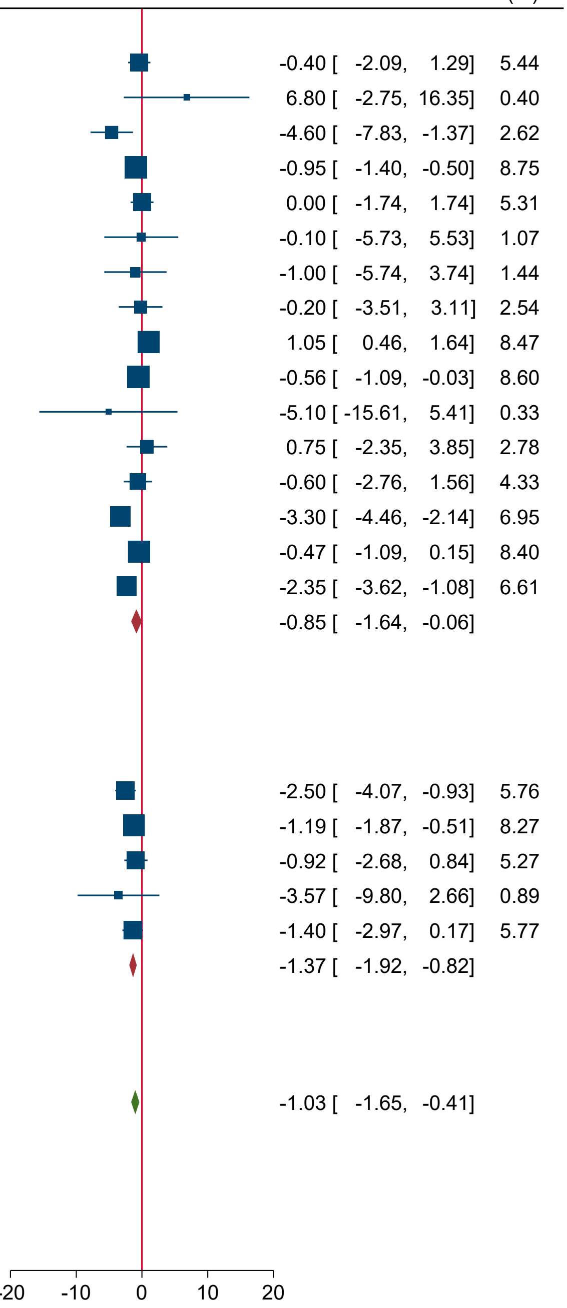

Random-effects REML model

Figure S2.14. The effect of lifestyle intervention in women with a history of gestational diabetes on body weight by intervention baseline mean age

Study	Treatment N	Treatment Mean	Treatment SD	Control N	Control Mean	Control SD	MD with 95% CI	Weight (%)
**Durng pregnancy**								
Lee 2022	130	65.6	15.8	168	65.8	13.3	-0.20 [-3.51, 3.11]	2.42
Quansah 2024	84	71.9	14.6	95	72.9	17.4	-1.00 [-5.74, 3.74]	1.37
Ferrara 2016	764	.39	5.5	875	.95	5.47	-0.56 [-1.09, -0.03]	8.19
Heterogeneity: $\tau^2 = 0.00$, $I^2 = 0.00\%$, $H^2 = 1.00$							-0.56 [-1.08, -0.03]	
Test of $\theta_i = \theta_j$: Q(2) = 0.08, p = 0.96								
Test of $\theta = 0$: z = -2.09, p = 0.04								
**Less than one year**								
McIntyre 2012	14	.97	3.7	11	.22	4.2	0.75 [-2.35, 3.85]	2.65
Geng 2014	50	60.7	5.2	50	61.1	3.2	-0.40 [-2.09, 1.29]	5.19
Sheng 2012	68	4.75	1.82	62	3.7	1.62	1.05 [0.46, 1.64]	8.06
Zilberman-Kravits 2018	60	73.11	16.11	44	76.68	15.91	-3.57 [-9.80, 2.66]	0.85
Cheung 2019	29	-1.7	4.1	20	-1.1	3.3	-0.60 [-2.76, 1.56]	4.12
McManus 2018	50	94.9	26	47	88.1	21.6	6.80 [-2.75, 16.35]	0.38
Holmes 2018	20	-3.9	7	25	.7	3.9	-4.60 [-7.83, -1.37]	2.50
O'Reilly 2016	206	-.23	2.48	228	.72	2.33	-0.95 [-1.40, -0.50]	8.34
Nicklas 2014	33	-2.8	2.04	35	.5	2.75	-3.30 [-4.46, -2.14]	6.62
Reinhardt 2012	15	74.1	16.3	16	79.2	13.5	-5.10 [-15.61, 5.41]	0.32
Tsoi 2024	40	-.93	4.68	39	-.01	3.12	-0.92 [-2.68, 0.84]	5.02
Heterogeneity: $\tau^2 = 2.24$, $I^2 = 84.18\%$, $H^2 = 6.32$							-1.02 [-2.19, 0.16]	
Test of $\theta_i = \theta_j$: Q(10) = 63.35, p = 0.00								
Test of $\theta = 0$: z = -1.69, p = 0.09								
**Mixed postpartum**								
Liew 2023	28	-.03	.97	28	.44	1.37	-0.47 [-1.09, 0.15]	8.01
Peacock 2015	11	-2.5	1.4	12	0	2.3	-2.50 [-4.07, -0.93]	5.49
Cheung 2011	16	1.6	4.5	15	.2	4.6	1.40 [-1.80, 4.60]	2.53
O'Dea 2015	16	.84	4.93	20	.08	5.66	0.76 [-2.76, 4.28]	2.21
Rollo 2020	15	-1.6	1.76	14	.75	1.74	-2.35 [-3.62, -1.08]	6.29
Tandon 2022	800	.4	17.8	801	.4	17.8	0.00 [-1.74, 1.74]	5.06
Shyam 2013	39	64.3	12.2	38	64.4	13	-0.10 [-5.73, 5.53]	1.02
Kim 2012	21	-1.5	3.4	28	-.1	2.2	-1.40 [-2.97, 0.17]	5.50
Heterogeneity: $\tau^2 = 0.78$, $I^2 = 55.38\%$, $H^2 = 2.24$							-1.00 [-1.90, -0.09]	
Test of $\theta_i = \theta_j$: Q(7) = 15.10, p = 0.03								
Test of $\theta = 0$: z = -2.15, p = 0.03								
**One year or more**								
Hu 2012	192	-1.4	3.44	212	-.21	3.52	-1.19 [-1.87, -0.51]	7.88
Heterogeneity: $\tau^2 = 0.00$, $I^2 = .\%$, $H^2 = .$							-1.19 [-1.87, -0.51]	
Test of $\theta_i = \theta_j$: Q(0) = -0.00, p = .								
Test of $\theta = 0$: z = -3.43, p = 0.00								
**Overall**							-0.93 [-1.53, -0.32]	
Heterogeneity: $\tau^2 = 1.09$, $I^2 = 76.42\%$, $H^2 = 4.24$								
Test of $\theta_i = \theta_j$: Q(22) = 82.44, p = 0.00								
Test of $\theta = 0$: z = -3.01, p = 0.00								
Test of group differences: $Q_b(3) = 2.35$, p = 0.50								

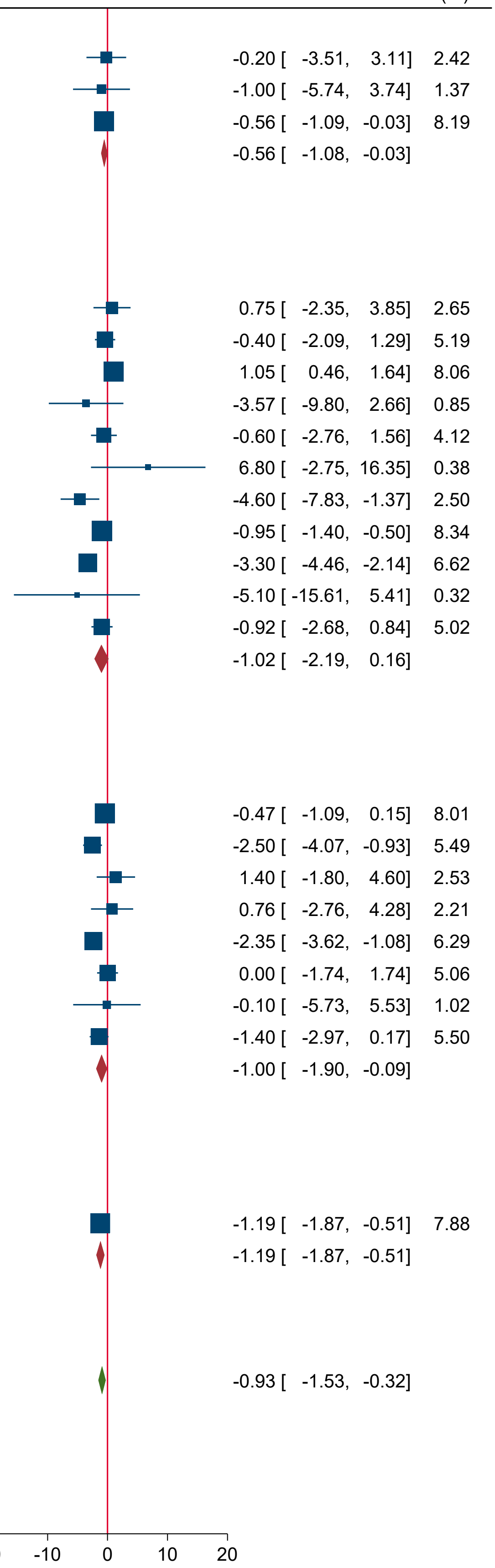

Random-effects REML model

Figure S2.15. The effect of lifestyle intervention in women with a history of gestational diabetes on body weight by intervention commencement time (Studies that described the time as less than *x* years or at least *y* months or a mean of *z* years were categorized as not explicitly stated).

	Treatment			Control				
Study	N	Mean	SD	N	Mean	SD	MD with 95% CI	Weight (%)
**No**								
Lee 2022	130	65.6	15.8	168	65.8	13.3	-0.20 [-3.51, 3.11]	2.42
Sheng 2012	68	4.75	1.82	62	3.7	1.62	1.05 [0.46, 1.64]	8.06
Reinhardt 2012	15	74.1	16.3	16	79.2	13.5	-5.10 [-15.61, 5.41]	0.32
Zilberman-Kravits 2018	60	73.11	16.11	44	76.68	15.91	-3.57 [-9.80, 2.66]	0.85
Geng 2014	50	60.7	5.2	50	61.1	3.2	-0.40 [-2.09, 1.29]	5.19
Tsoi 2024	40	-.93	4.68	39	-.01	3.12	-0.92 [-2.68, 0.84]	5.02
Liew 2023	28	-.03	.97	28	.44	1.37	-0.47 [-1.09, 0.15]	8.01
Tandon 2022	800	.4	17.8	801	.4	17.8	0.00 [-1.74, 1.74]	5.06
Kim 2012	21	-1.5	3.4	28	-.1	2.2	-1.40 [-2.97, 0.17]	5.50
O'Dea 2015	16	.84	4.93	20	.08	5.66	0.76 [-2.76, 4.28]	2.21
Hu 2012	192	-1.4	3.44	212	-.21	3.52	-1.19 [-1.87, -0.51]	7.88
Heterogeneity: $\tau^2 = 0.59$, $I^2 = 61.26\%$, $H^2 = 2.58$							-0.41 [-1.10, 0.28]	
Test of $\theta_i = \theta_j$: Q(10) = 31.08, p = 0.00								
Test of $\theta = 0$: z = -1.16, p = 0.25								
**Yes**								
Ferrara 2016	764	.39	5.5	875	.95	5.47	-0.56 [-1.09, -0.03]	8.19
Cheung 2019	29	-1.7	4.1	20	-1.1	3.3	-0.60 [-2.76, 1.56]	4.12
O'Reilly 2016	206	-.23	2.48	228	.72	2.33	-0.95 [-1.40, -0.50]	8.34
McManus 2018	50	94.9	26	47	88.1	21.6	6.80 [-2.75, 16.35]	0.38
Holmes 2018	20	-3.9	7	25	.7	3.9	-4.60 [-7.83, -1.37]	2.50
McIntyre 2012	14	.97	3.7	11	.22	4.2	0.75 [-2.35, 3.85]	2.65
Nicklas 2014	33	-2.8	2.04	35	.5	2.75	-3.30 [-4.46, -2.14]	6.62
Shyam 2013	39	64.3	12.2	38	64.4	13	-0.10 [-5.73, 5.53]	1.02
Rollo 2020	15	-1.6	1.76	14	.75	1.74	-2.35 [-3.62, -1.08]	6.29
Peacock 2015	11	-2.5	1.4	12	0	2.3	-2.50 [-4.07, -0.93]	5.49
Cheung 2011	16	1.6	4.5	15	.2	4.6	1.40 [-1.80, 4.60]	2.53
Quansah 2024	84	71.9	14.6	95	72.9	17.4	-1.00 [-5.74, 3.74]	1.37
Heterogeneity: $\tau^2 = 1.32$, $I^2 = 77.07\%$, $H^2 = 4.36$							-1.40 [-2.32, -0.49]	
Test of $\theta_i = \theta_j$: Q(11) = 36.19, p = 0.00								
Test of $\theta = 0$: z = -3.00, p = 0.00								
**Overall**							-0.93 [-1.53, -0.32]	
Heterogeneity: $\tau^2 = 1.09$, $I^2 = 76.42\%$, $H^2 = 4.24$								
Test of $\theta_i = \theta_j$: Q(22) = 82.44, p = 0.00								
Test of $\theta = 0$: z = -3.01, p = 0.00								
Test of group differences: $Q_b(1) = 2.87$, p = 0.09								

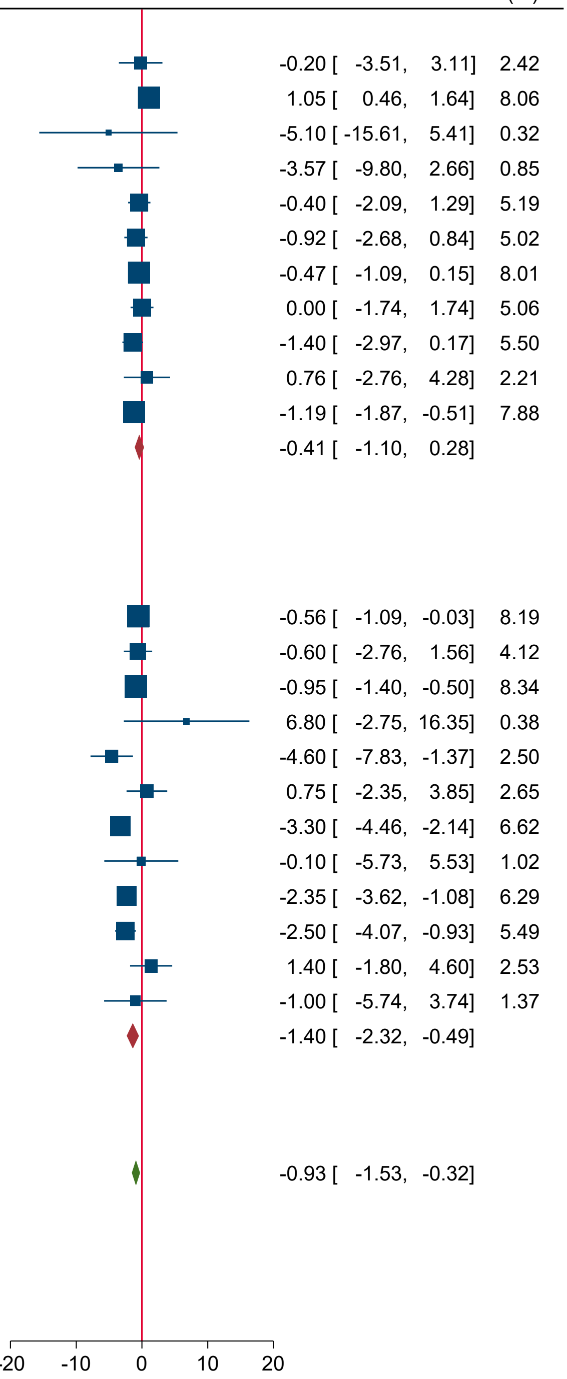

Random-effects REML model

Figure S2.16. The effect of lifestyle intervention in women with a history of gestational diabetes on body weight (theory-based vs non-theory-based interventions).

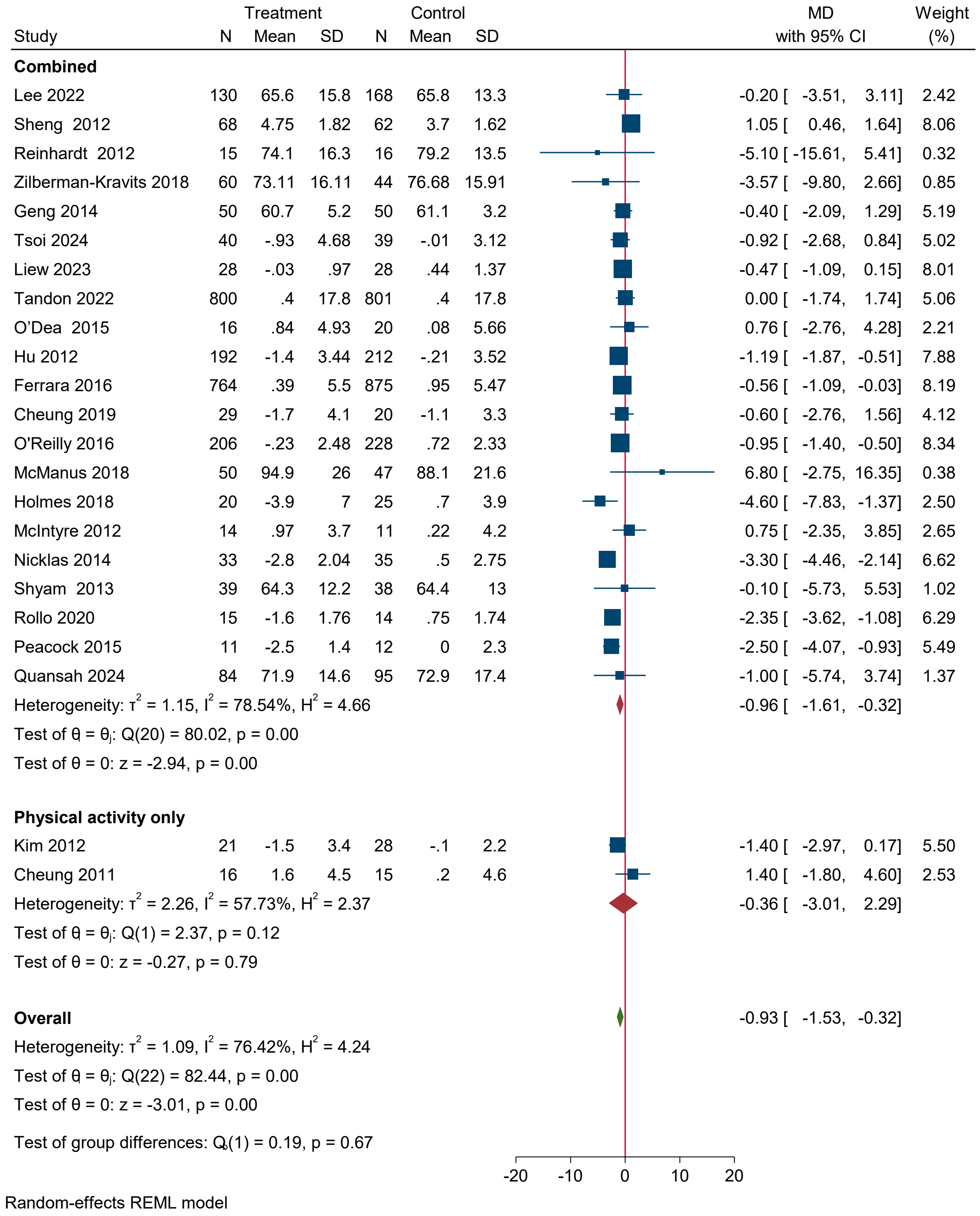

Figure S2.17. The effect of lifestyle intervention in women with a history of gestational diabetes on body weight by intervention type

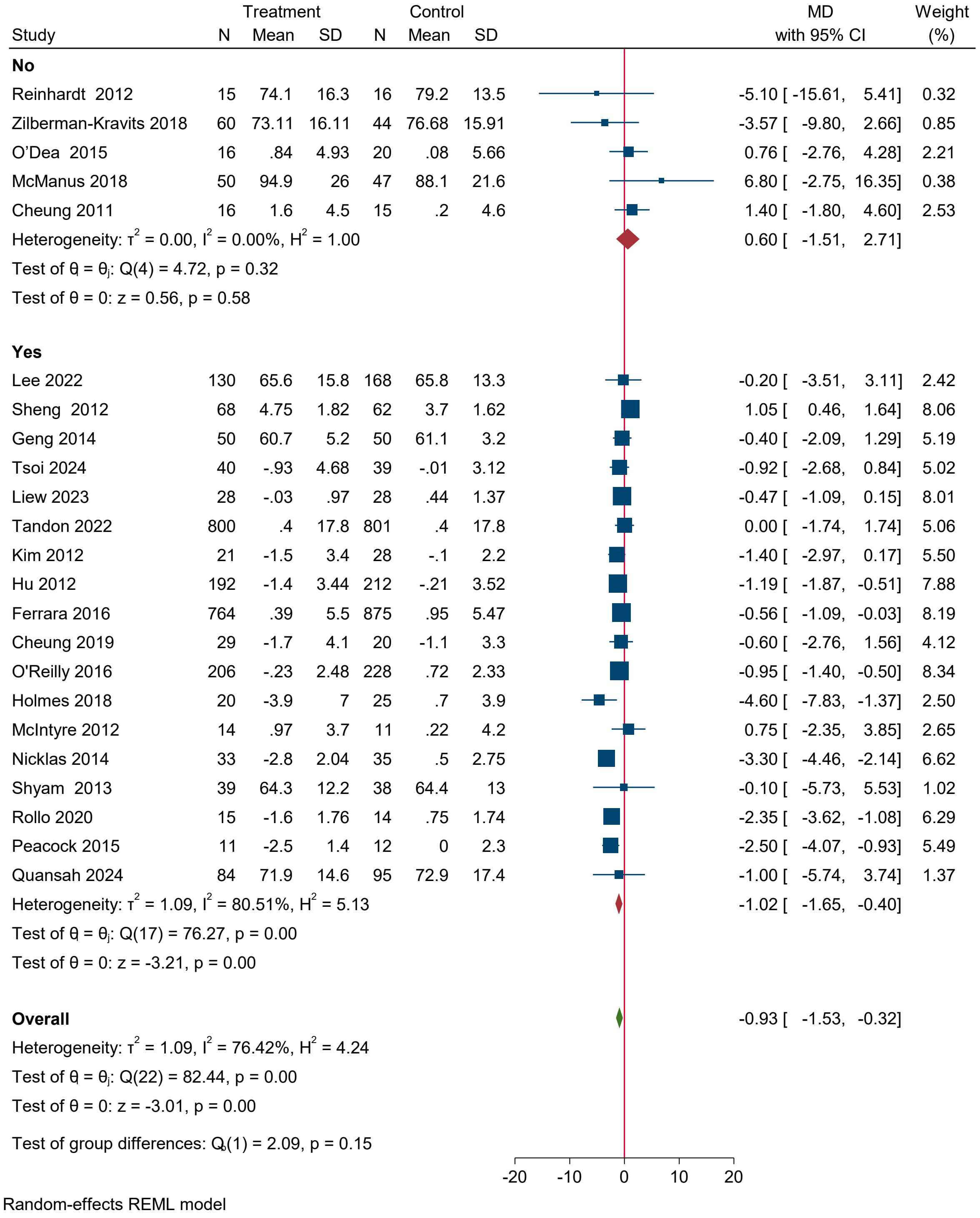

Figure S2.18. The effect of lifestyle intervention in women with a history of gestational diabetes on body weight (tailored vs non-tailored interventions).

Study	Treatment N	Treatment Mean	Treatment SD	Control N	Control Mean	Control SD	MD with 95% CI	Weight (%)
**In-person, face-to-face**								
Lee 2022	130	65.6	15.8	168	65.8	13.3	-0.20 [-3.51, 3.11]	2.42
Sheng 2012	68	4.75	1.82	62	3.7	1.62	1.05 [0.46, 1.64]	8.06
Zilberman-Kravits 2018	60	73.11	16.11	44	76.68	15.91	-3.57 [-9.80, 2.66]	0.85
O'Dea 2015	16	.84	4.93	20	.08	5.66	0.76 [-2.76, 4.28]	2.21
Heterogeneity: τ^2 = 0.00, I^2 = 0.00%, H^2 = 1.00							0.97 [0.39, 1.54]	
Test of $\theta_i = \theta_j$: Q(3) = 2.60, p = 0.46								
Test of θ = 0: z = 3.29, p = 0.00								
**In person + Telephone/message/website**								
Geng 2014	50	60.7	5.2	50	61.1	3.2	-0.40 [-2.09, 1.29]	5.19
McManus 2018	50	94.9	26	47	88.1	21.6	6.80 [-2.75, 16.35]	0.38
Holmes 2018	20	-3.9	7	25	.7	3.9	-4.60 [-7.83, -1.37]	2.50
O'Reilly 2016	206	-.23	2.48	228	.72	2.33	-0.95 [-1.40, -0.50]	8.34
Peacock 2015	11	-2.5	1.4	12	0	2.3	-2.50 [-4.07, -0.93]	5.49
Cheung 2011	16	1.6	4.5	15	.2	4.6	1.40 [-1.80, 4.60]	2.53
Tandon 2022	800	.4	17.8	801	.4	17.8	0.00 [-1.74, 1.74]	5.06
Shyam 2013	39	64.3	12.2	38	64.4	13	-0.10 [-5.73, 5.53]	1.02
Hu 2012	192	-1.4	3.44	212	-.21	3.52	-1.19 [-1.87, -0.51]	7.88
Quansah 2024	84	71.9	14.6	95	72.9	17.4	-1.00 [-5.74, 3.74]	1.37
Tsoi 2024	40	-.93	4.68	39	-.01	3.12	-0.92 [-2.68, 0.84]	5.02
Heterogeneity: τ^2 = 0.00, I^2 = 0.00%, H^2 = 1.00							-1.02 [-1.36, -0.69]	
Test of $\theta_i = \theta_j$: Q(10) = 15.16, p = 0.13								
Test of θ = 0: z = -5.93, p = 0.00								
**Telephone calls/messaging/mobile app**								
Liew 2023	28	-.03	.97	28	.44	1.37	-0.47 [-1.09, 0.15]	8.01
Ferrara 2016	764	.39	5.5	875	.95	5.47	-0.56 [-1.09, -0.03]	8.19
McIntyre 2012	14	.97	3.7	11	.22	4.2	0.75 [-2.35, 3.85]	2.65
Reinhardt 2012	15	74.1	16.3	16	79.2	13.5	-5.10 [-15.61, 5.41]	0.32
Cheung 2019	29	-1.7	4.1	20	-1.1	3.3	-0.60 [-2.76, 1.56]	4.12
Heterogeneity: τ^2 = 0.00, I^2 = 0.00%, H^2 = 1.00							-0.51 [-0.90, -0.12]	
Test of $\theta_i = \theta_j$: Q(4) = 1.42, p = 0.84								
Test of θ = 0: z = -2.54, p = 0.01								
**Web based**								
Nicklas 2014	33	-2.8	2.04	35	.5	2.75	-3.30 [-4.46, -2.14]	6.62
Rollo 2020	15	-1.6	1.76	14	.75	1.74	-2.35 [-3.62, -1.08]	6.29
Kim 2012	21	-1.5	3.4	28	-.1	2.2	-1.40 [-2.97, 0.17]	5.50
Heterogeneity: τ^2 = 0.40, I^2 = 46.81%, H^2 = 1.88							-2.45 [-3.50, -1.41]	
Test of $\theta_i = \theta_j$: Q(2) = 3.77, p = 0.15								
Test of θ = 0: z = -4.59, p = 0.00								
**Overall**							-0.93 [-1.53, -0.32]	
Heterogeneity: τ^2 = 1.09, I^2 = 76.42%, H^2 = 4.24								
Test of $\theta_i = \theta_j$: Q(22) = 82.44, p = 0.00								
Test of θ = 0: z = -3.01, p = 0.00								
Test of group differences: Q_b(3) = 46.74, p = 0.00								

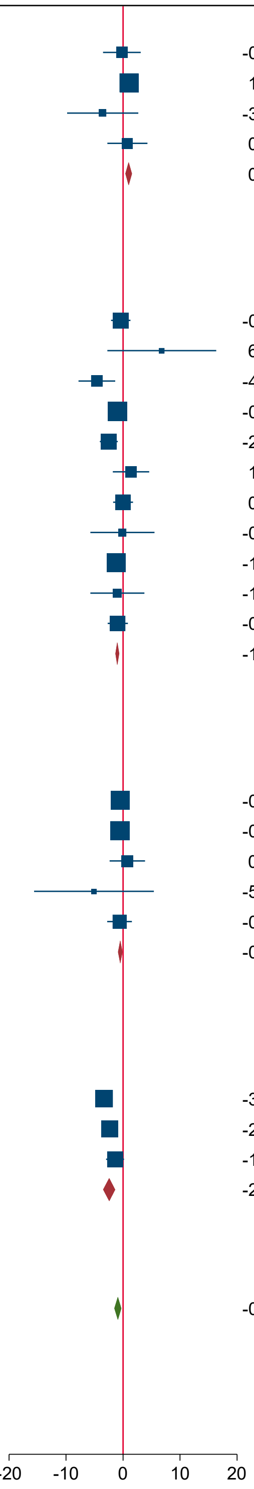

Random-effects REML model

Figure S2.19. The effect of lifestyle intervention in women with a history of gestational diabetes on body weight (face-to-face vs electronically delivered interventions)

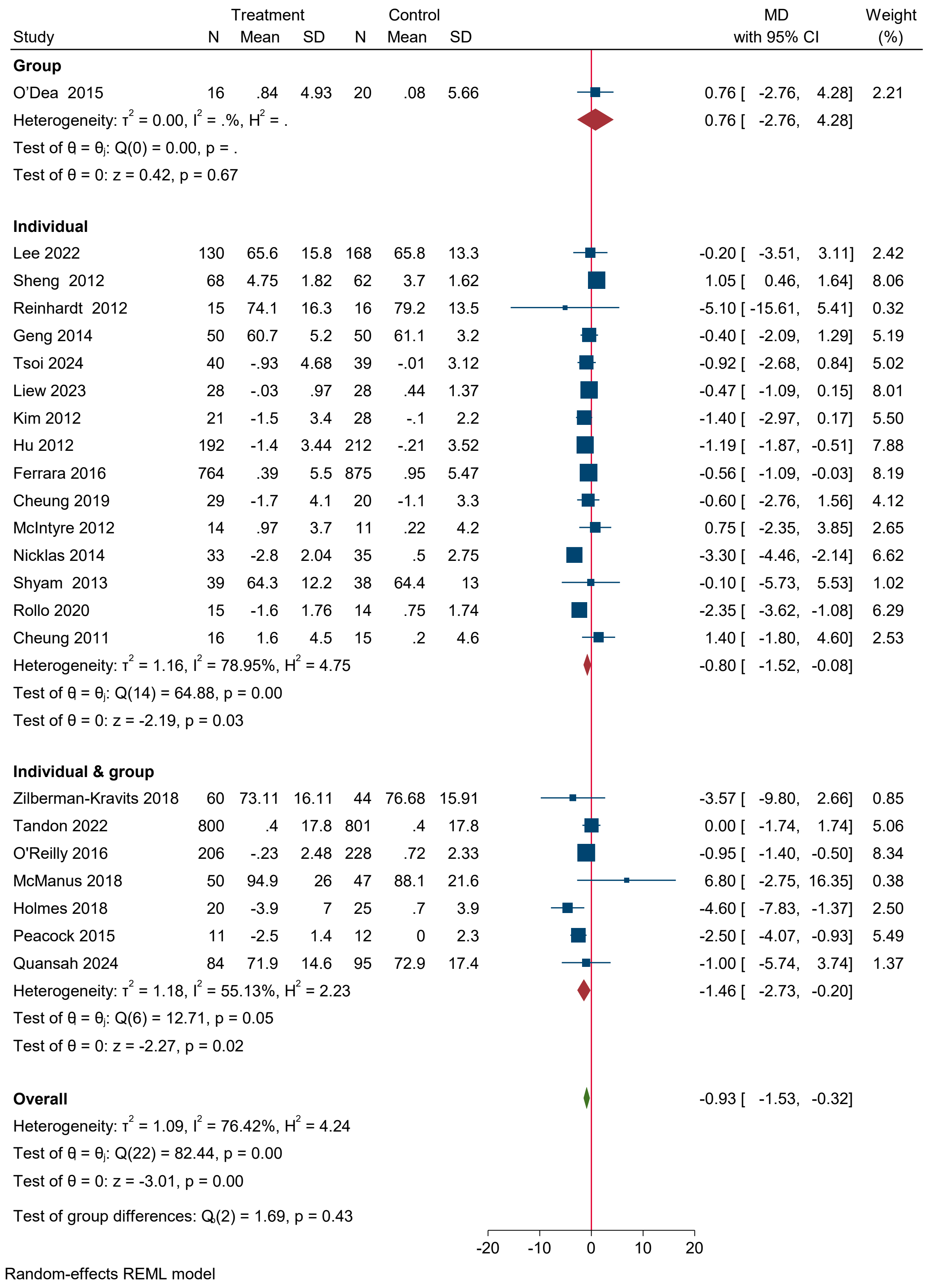
Treatment
Control
MD with 95% CI
Weight (%)
Study N Mean SD N Mean SD
Group
O'Dea 2015 16 .84 4.93 20 .08 5.66 0.76 [-2.76, 4.28] 2.21
Heterogeneity: τ² = 0.00, I² = .%, H² = . 0.76 [-2.76, 4.28]
Test of θi = θj: Q(0) = 0.00, p = .
Test of θ = 0: z = 0.42, p = 0.67
Individual
Lee 2022 130 65.6 15.8 168 65.8 13.3 -0.20 [-3.51, 3.11] 2.42
Sheng 2012 68 4.75 1.82 62 3.7 1.62 1.05 [0.46, 1.64] 8.06
Reinhardt 2012 15 74.1 16.3 16 79.2 13.5 -5.10 [-15.61, 5.41] 0.32
Geng 2014 50 60.7 5.2 50 61.1 3.2 -0.40 [-2.09, 1.29] 5.19
Tsoi 2024 40 -.93 4.68 39 -.01 3.12 -0.92 [-2.68, 0.84] 5.02
Liew 2023 28 -.03 .97 28 .44 1.37 -0.47 [-1.09, 0.15] 8.01
Kim 2012 21 -1.5 3.4 28 -.1 2.2 -1.40 [-2.97, 0.17] 5.50
Hu 2012 192 -1.4 3.44 212 -.21 3.52 -1.19 [-1.87, -0.51] 7.88
Ferrara 2016 764 .39 5.5 875 .95 5.47 -0.56 [-1.09, -0.03] 8.19
Cheung 2019 29 -1.7 4.1 20 -1.1 3.3 -0.60 [-2.76, 1.56] 4.12
McIntyre 2012 14 .97 3.7 11 .22 4.2 0.75 [-2.35, 3.85] 2.65
Nicklas 2014 33 -2.8 2.04 35 .5 2.75 -3.30 [-4.46, -2.14] 6.62
Shyam 2013 39 64.3 12.2 38 64.4 13 -0.10 [-5.73, 5.53] 1.02
Rollo 2020 15 -1.6 1.76 14 .75 1.74 -2.35 [-3.62, -1.08] 6.29
Cheung 2011 16 1.6 4.5 15 .2 4.6 1.40 [-1.80, 4.60] 2.53
Heterogeneity: τ² = 1.16, I² = 78.95%, H² = 4.75 -0.80 [-1.52, -0.08]
Test of θi = θj: Q(14) = 64.88, p = 0.00
Test of θ = 0: z = -2.19, p = 0.03
Individual & group
Zilberman-Kravits 2018 60 73.11 16.11 44 76.68 15.91 -3.57 [-9.80, 2.66] 0.85
Tandon 2022 800 .4 17.8 801 .4 17.8 0.00 [-1.74, 1.74] 5.06
O'Reilly 2016 206 -.23 2.48 228 .72 2.33 -0.95 [-1.40, -0.50] 8.34
McManus 2018 50 94.9 26 47 88.1 21.6 6.80 [-2.75, 16.35] 0.38
Holmes 2018 20 -3.9 7 25 .7 3.9 -4.60 [-7.83, -1.37] 2.50
Peacock 2015 11 -2.5 1.4 12 0 2.3 -2.50 [-4.07, -0.93] 5.49
Quansah 2024 84 71.9 14.6 95 72.9 17.4 -1.00 [-5.74, 3.74] 1.37
Heterogeneity: τ² = 1.18, I² = 55.13%, H² = 2.23 -1.46 [-2.73, -0.20]
Test of θi = θj: Q(6) = 12.71, p = 0.05
Test of θ = 0: z = -2.27, p = 0.02
Overall -0.93 [-1.53, -0.32]
Heterogeneity: τ² = 1.09, I² = 76.42%, H² = 4.24
Test of θi = θj: Q(22) = 82.44, p = 0.00
Test of θ = 0: z = -3.01, p = 0.00
Test of group differences: Qb(2) = 1.69, p = 0.43
-20 -10 0 10 20
Random-effects REML model

Figure S2.20. The effect of lifestyle intervention in women with a history of gestational diabetes on body weight by delivery mode (individual vs group).

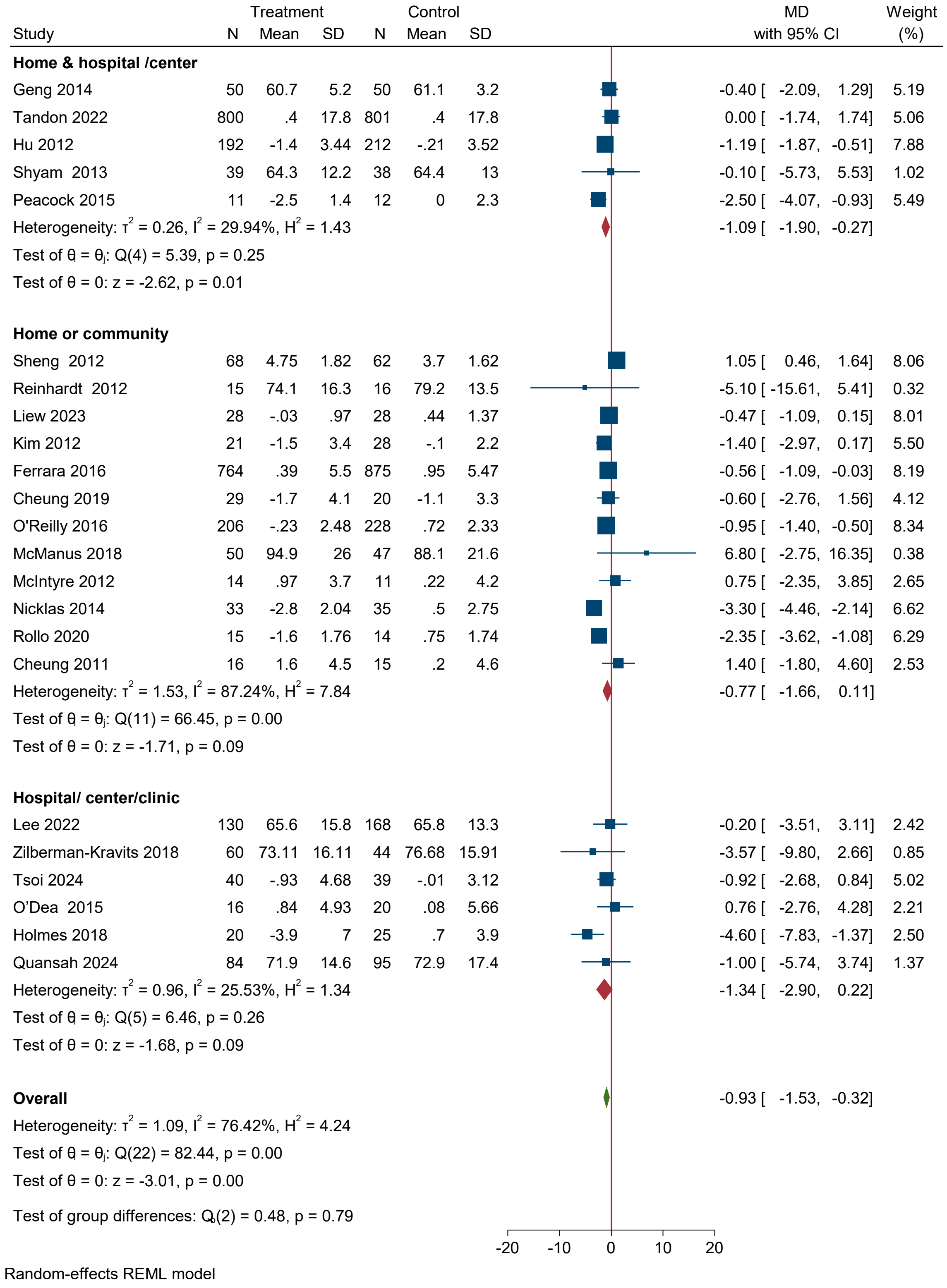

Figure S2.21. The effect of lifestyle intervention in women with a history of gestational diabetes on body weight by intervention location

Study	Treatment N	Treatment Mean	Treatment SD	Control N	Control Mean	Control SD	MD with 95% CI	Weight (%)
**< 3months**								
Liew 2023	28	-.03	.97	28	.44	1.37	-0.47 [-1.09, 0.15]	8.01
Cheung 2019	29	-1.7	4.1	20	-1.1	3.3	-0.60 [-2.76, 1.56]	4.12
McIntyre 2012	14	.97	3.7	11	.22	4.2	0.75 [-2.35, 3.85]	2.65
O'Dea 2015	16	.84	4.93	20	.08	5.66	0.76 [-2.76, 4.28]	2.21
Heterogeneity: τ^2 = 0.00, I^2 = 0.00%, H^2 = 1.00							-0.40 [-0.98, 0.18]	
Test of $\theta_i = \theta_j$: Q(3) = 1.03, p = 0.79								
Test of θ = 0: z = -1.37, p = 0.17								
**3 -12 months**								
Geng 2014	50	60.7	5.2	50	61.1	3.2	-0.40 [-2.09, 1.29]	5.19
Hu 2012	192	-1.4	3.44	212	-.21	3.52	-1.19 [-1.87, -0.51]	7.88
Shyam 2013	39	64.3	12.2	38	64.4	13	-0.10 [-5.73, 5.53]	1.02
Tandon 2022	800	.4	17.8	801	.4	17.8	0.00 [-1.74, 1.74]	5.06
Peacock 2015	11	-2.5	1.4	12	0	2.3	-2.50 [-4.07, -0.93]	5.49
Cheung 2011	16	1.6	4.5	15	.2	4.6	1.40 [-1.80, 4.60]	2.53
McManus 2018	50	94.9	26	47	88.1	21.6	6.80 [-2.75, 16.35]	0.38
O'Reilly 2016	206	-.23	2.48	228	.72	2.33	-0.95 [-1.40, -0.50]	8.34
Sheng 2012	68	4.75	1.82	62	3.7	1.62	1.05 [0.46, 1.64]	8.06
Reinhardt 2012	15	74.1	16.3	16	79.2	13.5	-5.10 [-15.61, 5.41]	0.32
Kim 2012	21	-1.5	3.4	28	-.1	2.2	-1.40 [-2.97, 0.17]	5.50
Ferrara 2016	764	.39	5.5	875	.95	5.47	-0.56 [-1.09, -0.03]	8.19
Nicklas 2014	33	-2.8	2.04	35	.5	2.75	-3.30 [-4.46, -2.14]	6.62
Rollo 2020	15	-1.6	1.76	14	.75	1.74	-2.35 [-3.62, -1.08]	6.29
Holmes 2018	20	-3.9	7	25	.7	3.9	-4.60 [-7.83, -1.37]	2.50
Tsoi 2024	40	-.93	4.68	39	-.01	3.12	-0.92 [-2.68, 0.84]	5.02
Heterogeneity: τ^2 = 1.42, I^2 = 83.40%, H^2 = 6.02							-1.10 [-1.85, -0.34]	
Test of $\theta_i = \theta_j$: Q(15) = 79.39, p = 0.00								
Test of θ = 0: z = -2.85, p = 0.00								
**>12 months**								
Quansah 2024	84	71.9	14.6	95	72.9	17.4	-1.00 [-5.74, 3.74]	1.37
Lee 2022	130	65.6	15.8	168	65.8	13.3	-0.20 [-3.51, 3.11]	2.42
Zilberman-Kravits 2018	60	73.11	16.11	44	76.68	15.91	-3.57 [-9.80, 2.66]	0.85
Heterogeneity: τ^2 = 0.00, I^2 = 0.00%, H^2 = 1.00							-0.96 [-3.44, 1.53]	
Test of $\theta_i = \theta_j$: Q(2) = 0.88, p = 0.65								
Test of θ = 0: z = -0.75, p = 0.45								
**Overall**							-0.93 [-1.53, -0.32]	
Heterogeneity: τ^2 = 1.09, I^2 = 76.42%, H^2 = 4.24								
Test of $\theta_i = \theta_j$: Q(22) = 82.44, p = 0.00								
Test of θ = 0: z = -3.01, p = 0.00								
Test of group differences: $Q_b(2)$ = 2.09, p = 0.35								

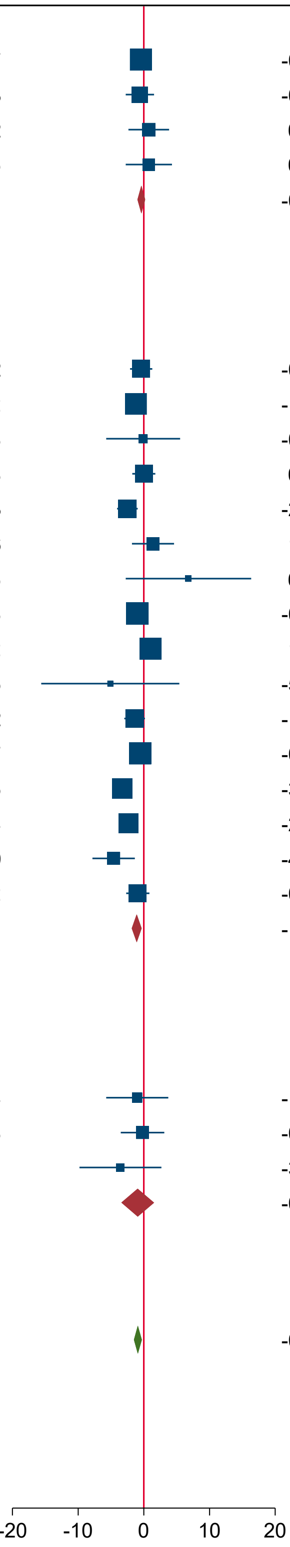

Random-effects REML model

Figure S2.22. The effect of lifestyle intervention in women with a history of gestational diabetes on body weight by intervention duration.

Study	Treatment N	Treatment Mean	Treatment SD	Control N	Control Mean	Control SD	MD with 95% CI	Weight (%)
**Low (1- 6.9)**								
Liew 2023	28	-.03	.97	28	.44	1.37	-0.47 [-1.09, 0.15]	8.01
McIntyre 2012	14	.97	3.7	11	.22	4.2	0.75 [-2.35, 3.85]	2.65
Geng 2014	50	60.7	5.2	50	61.1	3.2	-0.40 [-2.09, 1.29]	5.19
Peacock 2015	11	-2.5	1.4	12	0	2.3	-2.50 [-4.07, -0.93]	5.49
Cheung 2011	16	1.6	4.5	15	.2	4.6	1.40 [-1.80, 4.60]	2.53
Sheng 2012	68	4.75	1.82	62	3.7	1.62	1.05 [0.46, 1.64]	8.06
Ferrara 2016	764	.39	5.5	875	.95	5.47	-0.56 [-1.09, -0.03]	8.19
Holmes 2018	20	-3.9	7	25	.7	3.9	-4.60 [-7.83, -1.37]	2.50
Lee 2022	130	65.6	15.8	168	65.8	13.3	-0.20 [-3.51, 3.11]	2.42
Heterogeneity: τ^2 = 1.35, I^2 = 82.69%, H^2 = 5.78							-0.51 [-1.48, 0.46]	
Test of $\theta_i = \theta_j$: Q(8) = 36.10, p = 0.00								
Test of θ = 0: z = -1.03, p = 0.30								
**Medium (7-12)**								
O'Dea 2015	16	.84	4.93	20	.08	5.66	0.76 [-2.76, 4.28]	2.21
Hu 2012	192	-1.4	3.44	212	-.21	3.52	-1.19 [-1.87, -0.51]	7.88
Shyam 2013	39	64.3	12.2	38	64.4	13	-0.10 [-5.73, 5.53]	1.02
O'Reilly 2016	206	-.23	2.48	228	.72	2.33	-0.95 [-1.40, -0.50]	8.34
Nicklas 2014	33	-2.8	2.04	35	.5	2.75	-3.30 [-4.46, -2.14]	6.62
Rollo 2020	15	-1.6	1.76	14	.75	1.74	-2.35 [-3.62, -1.08]	6.29
Zilberman-Kravits 2018	60	73.11	16.11	44	76.68	15.91	-3.57 [-9.80, 2.66]	0.85
Tsoi 2024	40	-.93	4.68	39	-.01	3.12	-0.92 [-2.68, 0.84]	5.02
Heterogeneity: τ^2 = 0.74, I^2 = 69.27%, H^2 = 3.25							-1.58 [-2.43, -0.73]	
Test of $\theta_i = \theta_j$: Q(7) = 18.67, p = 0.01								
Test of θ = 0: z = -3.63, p = 0.00								
**High (>12)**								
Quansah 2024	84	71.9	14.6	95	72.9	17.4	-1.00 [-5.74, 3.74]	1.37
Cheung 2019	29	-1.7	4.1	20	-1.1	3.3	-0.60 [-2.76, 1.56]	4.12
Tandon 2022	800	.4	17.8	801	.4	17.8	0.00 [-1.74, 1.74]	5.06
McManus 2018	50	94.9	26	47	88.1	21.6	6.80 [-2.75, 16.35]	0.38
Reinhardt 2012	15	74.1	16.3	16	79.2	13.5	-5.10 [-15.61, 5.41]	0.32
Kim 2012	21	-1.5	3.4	28	-.1	2.2	-1.40 [-2.97, 0.17]	5.50
Heterogeneity: τ^2 = 0.00, I^2 = 0.00%, H^2 = 1.00							-0.70 [-1.70, 0.29]	
Test of $\theta_i = \theta_j$: Q(5) = 4.45, p = 0.49								
Test of θ = 0: z = -1.39, p = 0.17								
**Overall**							-0.93 [-1.53, -0.32]	
Heterogeneity: τ^2 = 1.09, I^2 = 76.42%, H^2 = 4.24								
Test of $\theta_i = \theta_j$: Q(22) = 82.44, p = 0.00								
Test of θ = 0: z = -3.01, p = 0.00								
Test of group differences: $Q_b(2)$ = 3.09, p = 0.21								

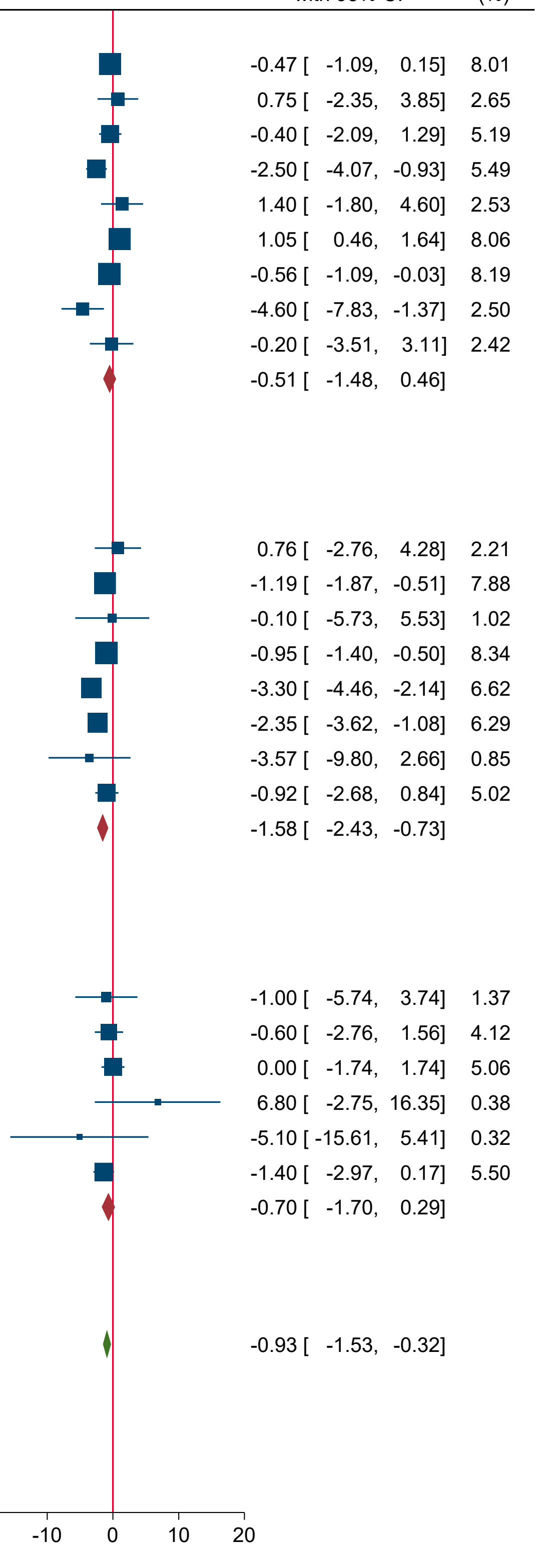

Random-effects REML model

Figure S2.23. The effect of lifestyle intervention in women with a history of gestational diabetes on body weight by number of sessions.

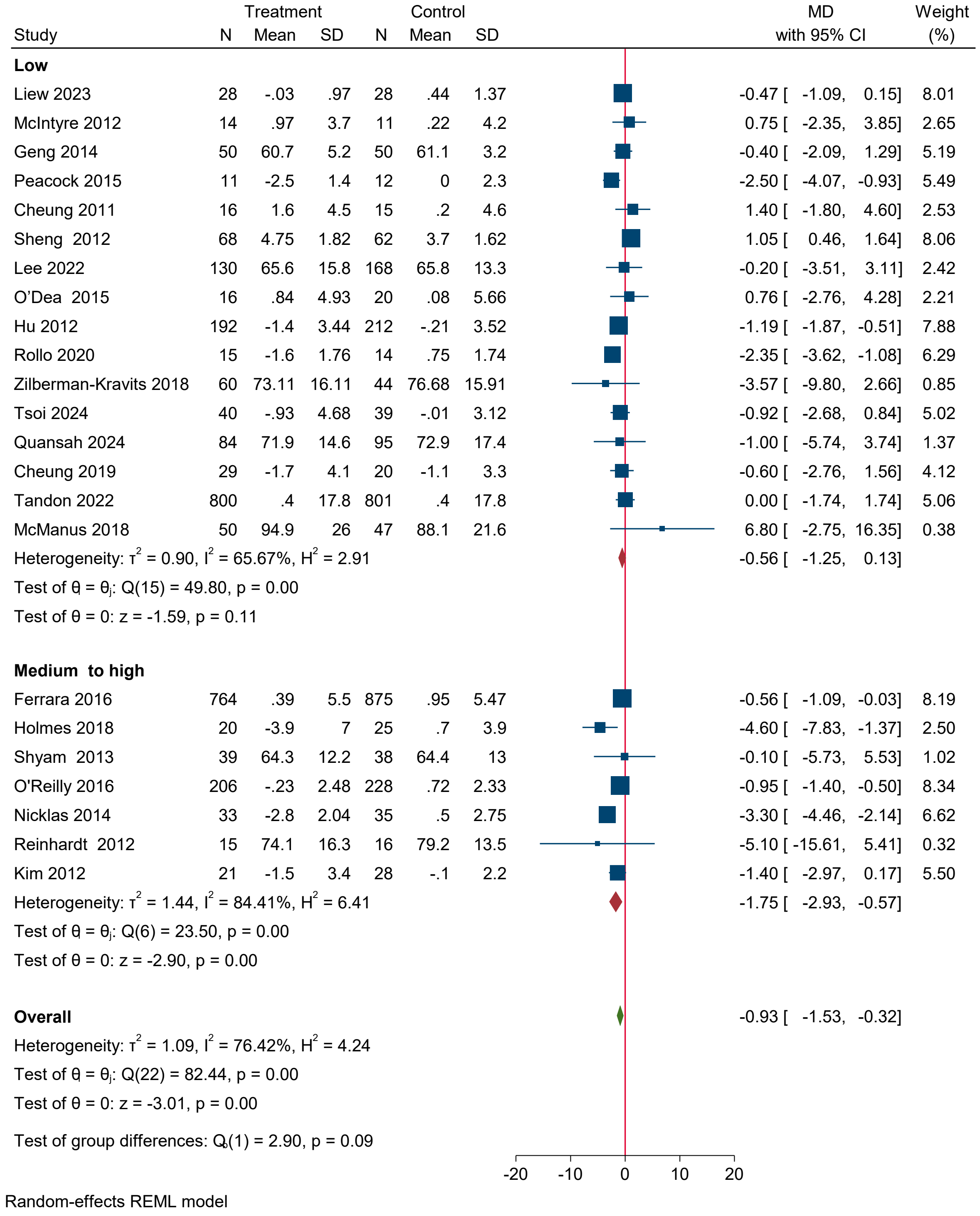

Figure S2.24. The effect of lifestyle intervention in women with a history of gestational diabetes on body weight by fidelity of the study.
